# Differential Dermatologic Adverse Events Associated With Checkpoint Inhibitor Monotherapy and Combination Therapy: A Meta-Analysis of Randomized Control Trials

**DOI:** 10.3389/fphar.2021.640099

**Published:** 2021-07-22

**Authors:** Yang Ge, Huiyun Zhang, Nathaniel Weygant, Jiannan Yao

**Affiliations:** ^1^ Beijing Chao-Yang Hospital, Dept. of Oncology, Capital Medical University, Beijing, China; ^2^ Academy of Integrative Medicine, Fujian Univ. of Traditional Chinese Medicine, Fuzhou, China; ^3^ Fujian Key Laboratory of Integrative Medicine in Geriatrics, Fuzhou, China

**Keywords:** meta-analysis, checkpoint inhibitors, combination immunotherapy, immune-related adverse events, dermatologic adverse events

## Abstract

**Background:** As immune checkpoint inhibitors (ICIs) transition to the forefront of cancer treatment, a better understanding of immune related adverse events (IRAEs) is essential to promote safe clinical practice. Dermatologic adverse events are the most common IRAEs and can lead to drug withdrawal and decreased quality of life. This meta-analysis aimed to investigate the risk of the most prevalent dermatologic adverse events (pruritus and rash) among various ICI treatment regimens.

**Methods:** A systematic search of electronic databases was performed to identify qualified randomized controlled trials (RCTs). Data for any grade and high grade pruritus and rash were extracted for meta-analysis. Two reviewers independently assessed methodological quality. The relative risk summary and 95% confidence interval were calculated.

**Results:** 50 RCTs involving 29941 patients were analyzed. The risk of pruritus (2.15 and 4.21 relative risk respectively) and rash (1.61 and 3.89 relative risk respectively) developing from CTLA-4 or PD-1/-L1 inhibitor were increased compared to placebo, but this effect was not dose-dependent. PD-1/-L1 plus CTLA-4 inhibitor was associated with increased risk of pruritus (1.76 and 0.98 relative risk respectively) and rash (1.72 and 1.37 relative risk respectively) compared to either monotherapy. Compared with CTLA-4 inhibitor, PD-1/-L1 inhibitor had a significantly decreased risk of pruritus and rash in both monotherapy and combination therapy (0.65 and 0.29 relative risk respectively). No significant difference was found between PD-1/-L1 inhibitor combined with chemotherapy and PD-1/-L1 monotherapy in any grade and high grade rash (0.84 and 1.43 relative risk respectively). In subgroup analyses, PD-1 inhibitor was associated with reduced risk of pruritus and rash compared to PD-L1 inhibitor.

**Conclusion:** Our meta-analysis demonstrates a better safety profile for PD-1/-L1 inhibitor compared to CTLA-4 inhibitor in terms of pruritus and rash among both monotherapy and multiple combination therapies. PD-L1 inhibitor may contribute to an increased risk of pruritus and rash compared to PD-1 inhibitor.

## Introduction

The application of immune checkpoint inhibitors (ICIs) is a significant milestone for clinical strategies in cancer. Due to increased activation of the immune system, ICIs can cause a spectrum of IRAEs that affect multiple organ systems and can even lead to death (Fausto et al., 2020). Dermatologic toxicities appear to be the most prevalent IRAEs, both with Programmed cell death protein 1/Programmed cell death-ligand 1 (PD-1/PD-L1) inhibitor and Cytotoxic T lymphocyte associate protein 4 (CTLA-4) inhibitor, and occur in more than a third of patients treated with ICI monotherapy (Sibaud et al., 2016). Consequently, decreased quality of life due to dermatologic adverse events may contribute to unnecessary drug withdrawal by patients. Additionally, management of serious dermatologic adverse events, including oral and topical steroids, may result in reduced drug efficacy (Geisler et al., 2020). Among dermatologic IRAEs manifestations, pruritus and rash are the most common (Boutros et al., 2016; Ellis et al., 2020; Geisler et al., 2020). Indeed, clinical studies demonstrate that pruritus may occur in 11–21% of patients treated with anti-PD-1/-L1 inhibitor, 24.4–35.4% of patients treated with CTLA-4 inhibitor, and 33.2–47% of patients in dual CTLA-4/PD-1 blockade (Geisler et al., 2020; Nishijima et al., 2017; Sibaud et al., 2016). For rash, incidence ranges as high as 20% for patients receiving PD-1 inhibitor, 14–26% for patients receiving CTLA-4 inhibitor, and 28.4–55% for patients receiving dual anti-CTLA-4/PD-1 blockade therapy (Geisler et al., 2020; Sibaud et al., 2016). Therefore, to balance the benefits and risks among multiple ICI treatment patterns in clinical strategy, an improved understanding of dermatologic IRAEs is essential (Collins et al., 2017; Ellis et al., 2020).

Combination immunotherapy has become a popular treatment option due to its superior clinical efficacy. However, ICI combination therapy is associated with toxic effects resulting from unbalanced activation of the immune system (Da et al., 2020). As mentioned above, combination of anti-CTLA-4 and anti-PD-1 therapy is associated with more frequent, more severe, and earlier dermatologic IRAEs compared to monotherapy (Almutairi et al., 2020; Sibaud et al., 2016). However, few studies have assessed dermatologic adverse events resulting from various ICI treatment regimens. Although previous meta-analysis (Nishijima et al., 2017; Yang et al., 2019) evaluated the incidence of selected dermatologic and mucosal adverse effects associated with PD-1/-L1 inhibitors, the authors included chemotherapy and ipilimumab as the only control arms. Other studies investigated the incidence and risk of IRAEs (including dermatologic adverse events) due to ICI monotherapy and combination therapy (Almutairi et al., 2020; Velasco et al., 2017; Wang et al., 2021), yet the patients included in their analysis were limited to a single tumor such as melanoma or lung cancer. Moreover, direct comparisons of the risk of dermatologic IRAEs between combination therapy and ICI monotherapy are lacking due to a dearth of head-to-head clinical trials. Therefore, a better understanding of dermatologic adverse events in this context is still needed. In the current study, we focused on the two most common dermatologic adverse events, pruritus and rash (Braun et al., 2020; Golian et al., 2016), in patients receiving ICI monotherapies and combination therapies including chemotherapy, targeted therapy, and other ICI treatment regimens. All the data used in this meta-analysis are derived from published literature and clinical trials.

## Materials and Methods

### Search Strategy and Eligibility Criteria

Two investigators (Yang Ge and Hui-Yun Zhang) independently searched PubMed, Embase, Web of Science, and the Cochrane Library. The last search was performed on January 20, 2020. The following terms were used: (Nivolumab or Opdivo or ONO-4538 or ONO 4538 MDX-1106 or BMS-936558 or pembrolizumab or lambrolizumab or Keytruda or cemiplimab or Pidilizumab or camrelizumab or SHR-1210 or JS001 or sintilimab or Durvalumab or MEDI4736 or atezolizumab or avelumab or Bavencio or tremelimumab or ticilimumab or Ipilimumab) and (Carcinoma or Neoplasia or Tumor or Cancer or Malignancy) and randomized controlled trials.

The following inclusion criteria were used: 1) studies included either ICI monotherapy or ICI combination therapy with chemotherapy/targeted therapy/ICIs in patients diagnosed with solid tumor; 2) studies investigated the following dermatologic adverse events: pruritus and rash; 3) randomized controlled clinical trials published in English. The following exclusion criteria were used: 1) phase I clinical trials; 2) studies without related data; 3) studies reporting dermatologic adverse events which are not related to ICIs; 3) editorials, letters, case reports, expert opinions, or reviews; and 4) duplicate publications.

### Data Extraction and Quality Assessment

The following information was extracted from each eligible study: first author, publication year, number of patients, cancer type, National Clinical Trial (NCT) number, randomization, trial phase, line of therapy, treatment, events of pruritus and rash in intervention and control arms (any grade and high grade). Our identification of any grade and high grade IRAEs was based on the Common Terminology Criteria for Adverse Events (CTCAE): “any grade” referred to CTCAE grades 1–5; “low grade” referred to CTCAE grades 1–2; “high grade” referred to CTCAE grades 3–5. The dosage of ICIs was also extracted to investigate if high dose ICIs are associated with increased IRAEs. Less than or equal to 3 mg/kg of PD-1/CTLA-4 was identified as “low dose”, while greater than or equal to 10 mg/kg was identified as “high dose”. The extraction was performed by two investigators (Yang Ge and Huiyun Zhang) independently and any controversies were resolved by discussion.

Quality assessment was performed using Review Manager 5.3. Risk of bias for the eligible study was evaluated according to following items recommended by the Cochrane Collaboration: randomization, allocation concealment blinding of participant, blinding of outcome assessors, incomplete outcome data, selective reporting, and other bias.

### Statistical Analysis

We conducted the meta-analysis using Review Manager 5.3. Risk ratio (RR) and 95% confidence interval (95% CI) were applied to evaluate the risk of pruritus and rash for both experimental and control arms. Relative risk ratio (RRR) with 95% CIs between different treatment regimens were calculated using RRs and 95% CIs. Heterogeneity was tested by the I^2^ and Q test. When *p* > 0.1 and I^2^ ≤ 50%, it was considered to indicate no significant heterogeneity and the fixed-effect model was applied. Otherwise, the random-effects model was applied. Begg’s and Egger’s tests were performed using Stata 16.0 to estimate publication bias. Subgroup analyses were performed to explore the sources of heterogeneity according to the different ICI class and tumor types.

## Results

### Search Results and Study Characteristics

**FIGURE 1:**
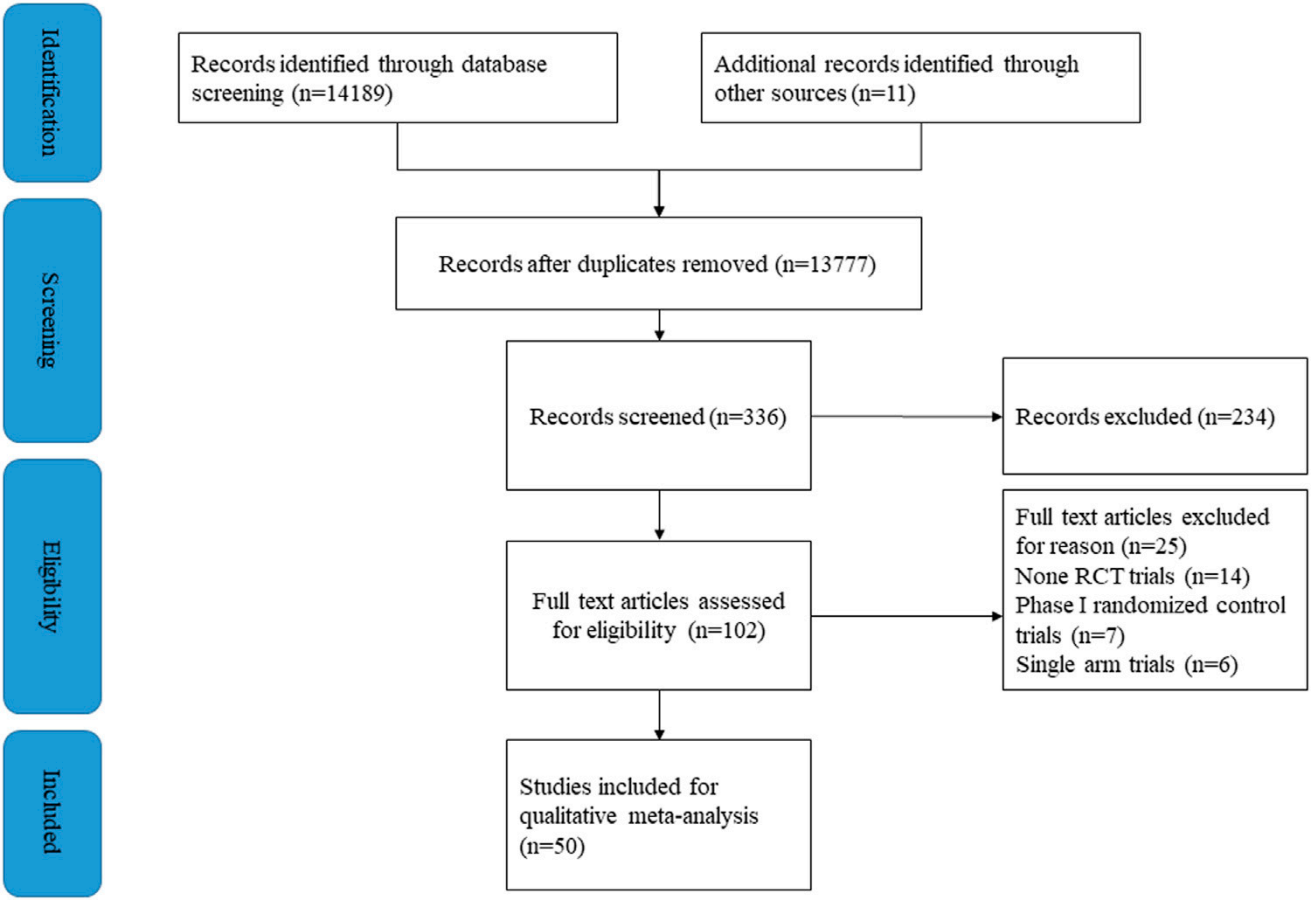
PRISMA flow chart of study selection.

14,819 publications were initially identified from the database and plus 11 from other sources. After excluding duplicates, 13,777 publications were assessed for review of title and abstract. 336 articles were further assessed for full-text review. Finally, 50 RCTs (*n* = 29,941 patients) were included in this meta-analysis **(** Figure 1 **)**. Most of the included studies involved patients with melanoma (*N* = 15) and none small cell lung carcinoma (NSCLC) (*N* = 12). The others were focused on renal cell carcinoma (RCC) (*N* = 5), head and neck squamous cell carcinoma (HNSCC) (*N* = 4), small cell lung cancer (SCLC) (*N* = 3), gastric cancer or gastro-oesophageal junction cancer (GC/GOJC) (*N* = 3), prostate cancer (*N* = 2), urothelial cancer (UC) (*N* = 2), malignant mesothelioma (*N* = 1), triple-negative breast cancer (TNBC) (*N* = 1), hepatocellular carcinoma (HCC) (*N* = 1), and pancreatic cancer (*N* = 1). Details of characteristics of the included studies are shown in Table 1.

**TABLE 1:** Characteristics of the included studies.

NCT	Author	Year	Cancer type	Phase	Line	Blinding	Treatment regimen	No. of patients	No. of pruritus events		No. of rash events	
									Any grade	High grade	Any grade	High grade
00289640	Wolchok et al. (2010)	2010	Melanoma	2	>1	Double-blind	Ipilimumab 10 mg/kg q3w	71	23	2	16	0
							Ipilimumab 3 mg/kg Q3w	71	15	1	17	1
							Ipilimumab 0.3 mg/kg Q3w	72	2	0	3	0
00324155	C. Robert et al. (2011)	2011	Melanoma	3	1	Double-blind	Ipilimumab (10 mg/kg) + dacarbazine (850 mg/m2 of body-surface area)given at weeks 1, 4, 7, and 10	247	66	5	55	3
							Placebo (10 mg/kg) + dacarbazine (850 mg/m2of body-surface area) given at weeks 1, 4, 7, and 10	251	15	0	12	0
00527735	Reck et al. (2013)	2013	SCLC	2	1	Double-blind	Ipilimumab plus chemotherapy	84	55	5	43	2
							Placebo plus chemotherapy	44	2	0	5	0
00257205	Ribas et al. (2013)	2013	Melanoma	3	1	None	Tremelimumab (15 mg/kg once every 90 days)	325	100	3	106	7
							Chemotherapy	319	16	0	17	1
00861614	Kwon et al. (2014)	2014	Prostate cancer	3	>1	Double-blind	Ipilimumab 10 mg/kg Q3W	393	80	1	68	2
							Placebo	396	15	0	16	0
01354431	Motzer et al. (2015b)	2015	Clear-cell renal cell carcinoma	2	>1	Double-blind	Nivolumab 0.3 mg/kg q3w	59	6	0	5	0
							Nivolumab 2 mg/kg q3w	54	5	1	4	0
							Nivolumab 10 mg/kg q3w	54	6	0	7	0
00636168	Eggermont et al. (2015)	2015	Melanoma	3	Adjuvant	Double-blind	Ipilimumab 10 mg/kg q3w	471	187	11	162	52
							Placebo	474	51	0	6	0
01642004	Brahmer et al. (2015)	2015	NSCLC	3	>1	None	Nivolumab 3 mg/kg Q2W	131	3	0	5	0
							Docetaxel 75 mg/m^2 Q3W	129	0	0	8	2
01668784	Motzer et al. (2015a)	2015	RCC	3	>1	None	Nivolumab 3 mg/kg Q2W	406	57	39	41	2
							Everolimus 10 mg QD	397	0	0	79	3
01673867	Borghaei et al. (2015)	2015	NSCLC	3	>1	None	Nivolumab 3 mg/kg Q2W	287	24	0	27	1
							Docetaxel 75 mg/m^2 Q3W	268	4	0	8	0
01704287	Ribas et al. (2015)	2015	Melanoma	2	>1	Double-blind	Pembrolizumab 10 mg/kg Q3w	179	42	0	18	0
							Pembrolizumab 2 mg/kg Q3w	178	37	0	21	0
							Chemotherapy	171	6	0	8	0
01721746	Weber et al. (2015)	2015	Melanoma	3	>1	None	Nivolumab	268	43	0	25	1
							Chemotherapy	102	2	0	5	0
01721772	Robert et al. (2015a)	2015	Melanoma	3	1	Double-blind	Nivolumab 3 mg/kg Q2W	206	35	1	31	1
							Dacarbazine 1,000 mg/m^2 Q3W	205	11	0	6	0
01844505	Larkin et al. (2015)	2015	Melanoma	3	1	Double-blind	Ipilimumab 3 mg/kg Q3W for four cycles	311	110	1	65	5
							Nivolumab 1 mg/kg + ipilimumab 3 mg/kg Q3W	313	104	6	89	9
							Nivolumab 3 mg/kg Q2W	313	59	0	68	1
01866319	Robert et al. (2015b)	2015	Melanoma	3	≥1	None	Ipilimumab 3 mg/kg Q3w	256	65	1	37	2
							Pembrolizumab 10 mg/kg Q2w	278	40	0	41	0
							Pembrolizumab 10 mg/kg Q3w	277	39	0	37	0
01927419	Postow et al. (2015b)	2015	Melanoma	2	1	Double-blind	Nivolumab 1 mg/kg + ipilimumab 3 mg/kg Q3W for four cycles	94	33	1	39	5
							Placebo 1 mg/kg + ipilimumab 3 mg/kg Q3W	46	13	0	12	0
01057810	Beer et al. (2016)	2016	Prostate cancer	3	1	Double-blind	Ipilimumab 10 mg/kg q3w	399	109	1	132	10
							Placebo	199	14	1	15	0
01450761	Reck et al. (2016)	2016	SCLC	3	1	None	Etoposide andplatinum (cisplatin or carboplatin) plus ipilimumab 10 mg/kg q3w	154	55	3	90	8
							Etoposide andplatinum (cisplatin or carboplatin) plus placebo 10 mg/kg q3w	150	8	0	12	0
01905657	Herbst et al. (2016)	2016	NSCLC	2/3	>1	None	Pembrolizumab 10 mg/kg, Q3w	343	32	0	44	1
							Pembrolizumab 2 mg/kg, Q3w	339	25	0	29	1
							Docetaxel 75 mg/m^2^ every 3 weeks	309	5	1	14	0
02039674	Langer et al. (2016)	2016	NSCLC	2	1	None	Pembrolizumab 200 mg + pemetrexed 500 mg/m^2^ + carboplatin area under curve 5 mg/ml q3w	59	7	0	16	1
							Pemetrexed 500 mg/m^2^ + carboplatin AUC 5 mg/ml per min	62	2	0	9	0
02105636	Ferris et al. (2016)	2016	HNC	3	>1	None	Nivolumab 3 mg/kg Q2W	236	17	0	18	0
							Standard therapy	111	0	0	5	1
01285609	Govindan et al. (2017)	2017	NSCLC	3	>1	Double-blind	Paclitaxel and carboplatin plus blinded ipilimumab 10 mg/kg q3w	388	56	4	67	8
							Placebo plus chemotherapy	361	8	0	14	0
01515189	Ascierto et al. (2017)	2017	Melanoma	3	≥1	Double-blind	Ipilimumab 3 mg/kg Q3w	362	82	2	95	5
							Ipilimumab 10 mg/kg Q3w	364	81	2	5	2
01843374	Maio et al. (2017)	2017	Malignant mesothelioma	2	>1	Double-blind	Tremelimumab 10 mg/kg Q4w	380	103	3	79	2
							Placebo	189	15	0	13	0
02041533	Carbone et al. (2017)	2017	NSCLC	3	1	None	Nivolumab 3 mg/kg Q2W	267	22	0	26	2
							Investigator’s choice chemotherapy Q3W	263	7	1	15	1
02125461	Antonia et al. (2017)	2017	NSCLC	3	>1	Double-blind	Durvalumab (10 mg per kilogram of body weight) q2w	475	33	0	37	1
							Placebo q2w	234	5	0	13	0
02256436	Bellmunt et al. (2017)	2017	UC	3	>1	None	Pembrolizumab 200 mg q3w	266	52	0	NA	NA
							Chemotherapy	255	7	1	NA	NA
02267343	Kang et al. (2017)	2017	GC/GOJC	3	>1	Double-blind	3 mg/kg nivolumab Q2W	330	30	0	19	5
							Placebo	161	9	0	0	0
02388906	Weber et al. (2017)	2017	Melanoma	3	1	Double-blind	Ipilimumab 10 mg/kg Q3W	453	152	5	133	14
							Nivolumab 3 mg/kg Q2W	452	105	0	90	5
01928394	Janjigian et al. (2018)	2018	Esophagogastric cancer	2	>1	None	Nivolumab 3 mg/kg + ipilimumab 1 mg/kg Q3W	52	12	0	8	0
							Nivolumab 1 mg/kg + ipilimumab 3 mg/kg Q3W	49	9	1	10	0
							Nivolumab 3 mg/kg Q2W	59	10	0	5	0
02302807	Powles et al. (2018)	2018	Urothelial bladder cancer	3	>1	None	Atezolizumab 1,200 mg Q3W	459	59	NA	40	NA
							Chemotherapy	443	14	NA	21	NA
02362594	Eggermont et al. (2018)	2018	Melanoma	3	Adjuvant	Double-blind	Pembrolizumab 200 mg q3w	509	90	0	82	1
							Placebo	502	51	0	52	0
02366143	Socinski et al. (2018)	2018	NSCLC	3	1	None	Atezolizumab 1,200 mg plus bevacizumab plus carboplatin plus paclitaxel	393	NA	NA	52	5
							Bevacizumab plus carboplatin plus paclitaxel	394	NA	NA	20	0
02374242	Long et al. (2018)	2018	Melanoma	2	≥1	None	Nivolumab 1 mg/kg + ipilimumab 3 mg/kg q3w	35	13	0	22	4
							Nivolumab 3 mg/kg q2w	25	2	0	5	0
02425891	Schmid et al. (2018)	2018	TNBC	3	1	Double-blind	Atezolizumab plus nab-paclitaxel	452	46	0	59	2
							Placebo plus nab-paclitaxel	438	36	0	54	2
02477826	Hellmann et al. (2018)	2018	Lung cancer	3	1	None	Nivolumab 3 mg/kg Q2w + ipilimumab 1 mg/kg Q6W	576	81	3	96	9
							Nivolumab 240 mg Q2W	391	30	0	43	3
			Chemotherapy			570	5	0	29	0
02578680	Gandhi et al. (2018)	2018	NSCLC	3	1	Double-blind	Pembrolizumab 200 mg q3w + carboplatin/cisplatin 75 mg/kg/m2 q3w + pemetrexed 5 mg/kg/m2 q3w	405	55	NA	109	NA
							placebo200 mg q3w + carboplatin/cisplatin 75 mg/kg/m2 q3w + pemetrexed 5 mg/kg/m2 q3w	202	22	NA	28	NA
02763579	Horn et al. (2018)	2018	SCLC	3	1	Double-blind	Atezolizumab plus chemotherapy	198	NA	NA	37	4
							Placebo plus chemotherapy	196	NA	NA	20	0
02775435	Paz-Ares et al. (2018)	2018	NSCLC	3	1	Double-blind	Pembrolizumab plus chemotherapy	278	40	NA	47	NA
							Placebo plus chemotherapy	280	25	NA	32	NA
02220894	Mok et al. (2019)	2019	NSCLC	3	1	None	Pembrolizumab 200 mg q3w	636	46	2	46	3
							Chemotherapy	615	15	0	27	0
02252042	Cohen et al. (2019)	2019	HNC	3	>1	None	Pembrolizumab 200 mg q3w	246	12	0	19	1
							Chemotherapy	234	16	2	34	1
02358031	Burtness et al. (2019)	2019	HNSCC	3	1	None	Pembrolizumab 200 mg every 3 weeks	330	NA	NA	25	2
							Pembrolizumab 200 mg every 3 weeks + carboplatin (5 mg/m^2^)/cisplatin (100 mg/m^2^) + 5-fluorouracil (1,000 mg/m^2^ per day for 4 consecutive days) q3w	276	NA	NA	23	1
							.Cetuximab (400 mg/m^2^ loading dose, then 250 mg/m^2^ qw)+carboplatin (5 mg/m^2^)/cisplatin (100 mg/m^2^) + 5-fluorouracil (1,000 mg/m^2^ per day for 4 consecutive days) q3w	287	NA	NA	101	17
02319044	Siu et al. (2019)	2019	HNSCC	2	>1	None	Durvalumab 20 mg/kg Q4w plus tremelimumab 1 mg/kg Q4w for 4 cycles, durvalumab 10 mg/kg Q2W	133	5	NA	9	NA
							Durvalumab 10 mg/kg Q2w for 4 cycles, durvalumab 10 mg/kg Q2W	65	5	NA	1	NA
							Tremelimumab 10 mg/kg Q4w for 7 cycles, tremelimumab 10 mg/kg Q12w for 2 cycles	65	3	NA	5	NA
02420821	Rini et al. (2019b)	2019	RCC	3	1	None	Atezolizumab 1200 mg plus bevacizumab 15 mg/kg Q3W	451	85	0	70	3
							Sunitinib 50 mg QD	446	22	0	53	2
02558894	O'Reilly et al. (2019)	2019	Pancreatic ductal carcinoma	2	>1	None	Durvalumab (1,500 mg every 4 weeks)	33	2	0	NA	NA
							Durvalumab (1,500 mg every 4 weeks) plus tremelimumab (75 mg every 4 weeks)	32	1	0	NA	NA
02569242	Kato et al. (2019)	2019	Oesophageal squamous cell carcinoma	3	>1	None	Nivolumab 240 mg Q2W	209	NA	NA	23	1
							Chemotherapy	208	NA	NA	31	2
02684006	Motzer et al. (2019)	2019	RCC	3	1	None	Avelumab (10 mg per kilogram of body weight) q2w + axitinib (5 mg) orally twice daily	434	53	0	54	2
							Sunitinib (50 mg) orally once daily	439	19	0	42	2
02702401	Finn. et al. (2019)	2019	HCC	3	>1	Double-blind	Pembrolizumab 200 mg q3w	279	37	1	23	1
							Placebo	134	6	0	3	0
02714218	Celeste et al. (2019)	2019	Melanoma	3	1	Double-blind	Nivolumab 1 mg/kg + ipilimumab 3 mg/kg Q3W	178	47	0	47	0
							Nivolumab 3 mg/kg + ipilimumab 1 mg/kg Q3W	180	43	1	31	0
02853331	Rini et al. (2019a)	2019	RCC	3	1	None	Pembrolizumab plus axitinib	429	53	1	46	1
							Sunitinib	425	18	0	38	1

### Incidence of Pruritus/Rash Associated With Immune Checkpoint Inhibitor Monotherapy or Combination Therapy

#### Immune Checkpoint Inhibitors Monotherapy Vs Placebo

A total of four studies including 2,624 patients were assessed in this analysis. When comparing PD-1/-L1 inhibitor with placebo, the RR was 2.15 (95% CI 1.60-2.89, *p* < 0.00001) (Supplementary Figure 1A) for any grade pruritus. For high grade pruritus, RR could not be assessed because less than 3 RCTs were available. For rash, the RRs were 1.61 (95% CI 1.24-2.11, *p* = 0.0004) (Supplementary Figure 1B) and 1.87 (95% CI 0.30-11.56, *p* = 0.50), for any grade and high grade respectively (Supplementary Figure 1C). A similar result was found when comparing CTLA-4 inhibitor with placebo. The RRs were 4.21 (95% CI 3.48-5.10, *p* < 0.00001) (Supplementary Figure 2A) and 5.57 (95% CI 1.77-17.48, *p* = 0.003) (Supplementary Figure 2B) for any grade and high grade pruritus respectively. For rash, the RRs were 3.89 (95% CI 3.21-4.72, *p* < 0.00001) (Supplementary Figure 2C) and 7.37 (95% CI 2.24, 24.25, *p* = 0.001) for any grade and high grade respectively (Supplementary Figure 2D).

#### Programmed Cell Death Protein 1/Programmed Cell Death-Ligand 1 Inhibitor Vs Chemotherapy

**FIGURE 2:**
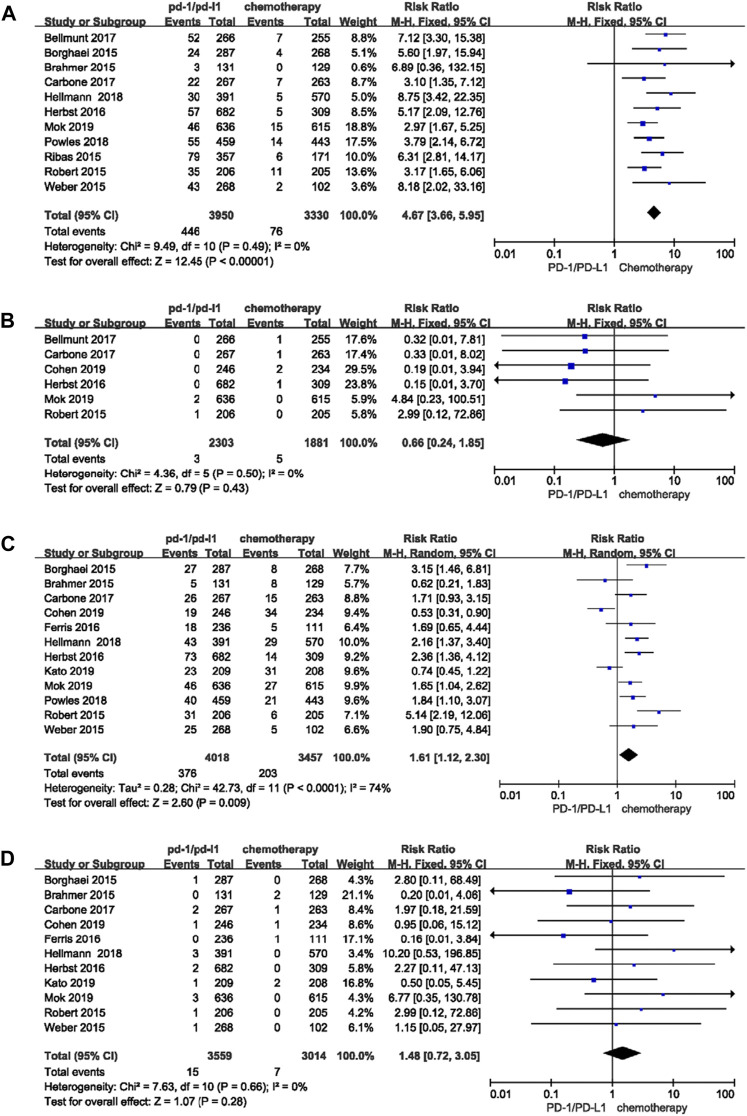
Forest plots of the relative risks and 95% CIs for pruritus and rash after PD-1/-L1 inhibitor compared to chemotherapy. **(A)** any grade pruritus; **(B)** high grade pruritus; **(C)** any grade rash; **(D)** high grade rash.

To make a comparison between PD-1/-L1 inhibitor and chemotherapy, 8,107 patients from 13 studies were included. The RRs for any grade and high grade pruritus were 4.67 (95% CI 3.66–5.95, *p* < 0.00001) (Figure 2A) and 0.66 (95% CI 0.24-1.85 *p* = 0.43), respectively (Figure 2B). For rash, the RRs were 1.61 (95% CI 1.12-2.30, *p* = 0.009) (Figure 2C) and 1.48 (95% CI 0.72-3.05, *p* = 0.28) (Figure 2D) for any grade and high grade, respectively.

#### Programmed Cell Death Protein 1/Programmed cell Death-Ligand 1 Vs CTLA-4 Inhibitor

To investigate the difference in pruritus and rash between PD-1/-L1 inhibitor and CTLA-4 inhibitor, four studies with 2,370 patients were included. RRs for any grade and high grade pruritus developed after PD-1/-L1 inhibitor treatment were 0.65 (95%CI 0.56-0.75, *p* < 0.00001) (Supplementary Figure 3A) and 0.15 (95%CI 0.03-0.89, *p* = 0.04) (Supplementary Figure 3B) respectively compared to CTLA-4 inhibitor treatment. For rash the RRs were 1.06 (95%CI 0.85-1.34, *p* = 0.60) (Supplementary Figure 3C) and 0.29 (95%CI 0.12-0.68, *p* = 0.005) for any grade and high grade respectively (Supplementary Figure 3D).

#### High Dose Vs Low Dose Programmed Cell Death Protein 1/Programmed Cell Death-Ligand 1 Inhibitor

In this section, five qualifying studies with 2,015 patients total were analyzed. Compared to low dose groups, RRs for any grade pruritus and any grade rash developed after high dose PD-1/PD-L1 inhibitor therapy were 0.84 (95%CI 0.63-1.14, *p* = 0.26) (Supplementary Figure 4A) and 0.79 (95%CI 0.56-1.11, *p* = 0.17) respectively (Supplementary Figure 4B).

#### Immune Checkpoint Inhibitors Combination Chemotherapy Vs Chemotherapy Alone

**FIGURE 3:**
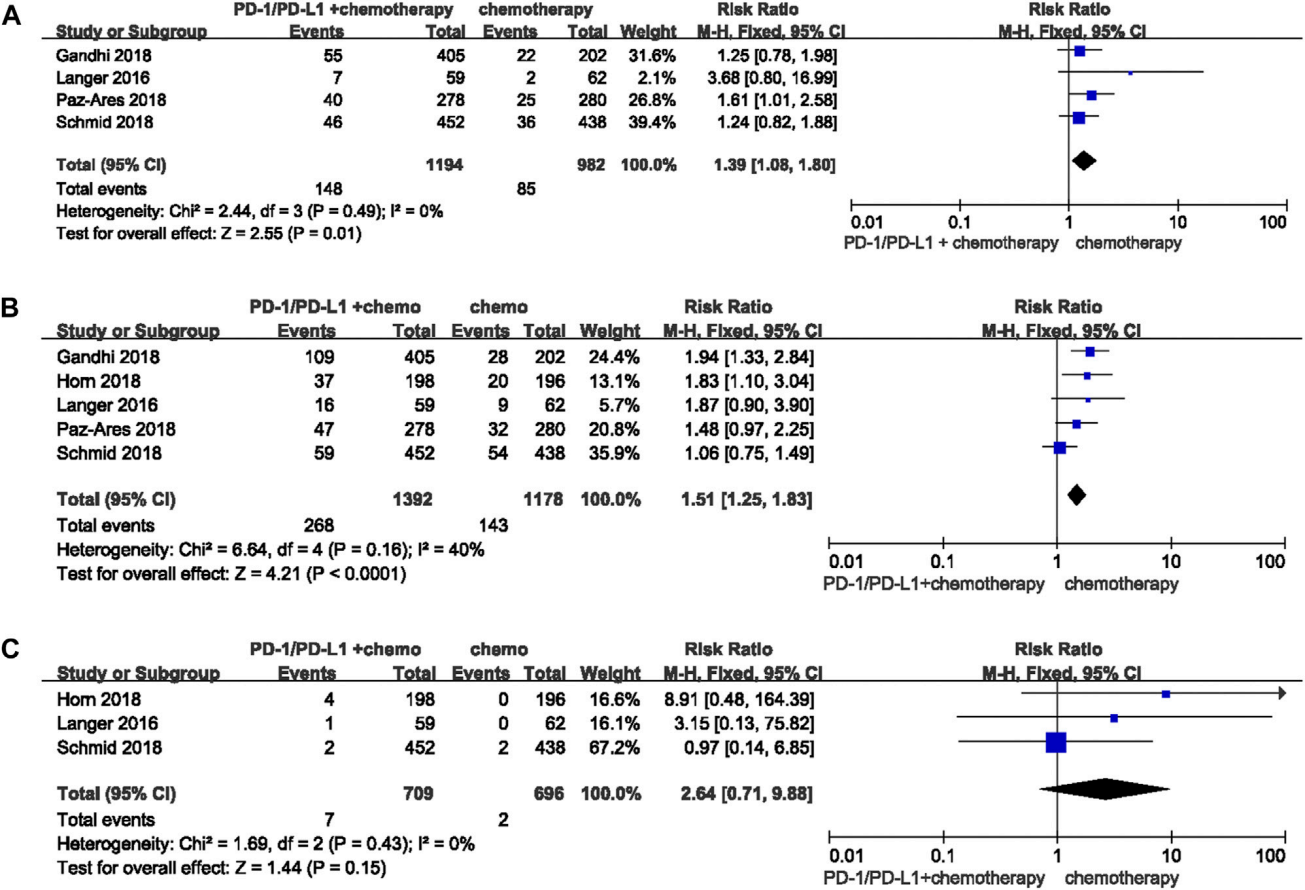
Forest plots of the relative risks and 95% CIs for pruritus and rash in comparison of PD-1/-L1 plus chenotherapy and chemotherapy. **(A)** any grade pruritus; **(B)** any grade rash; **(C)** high grade rash.

**FIGURE 4:**
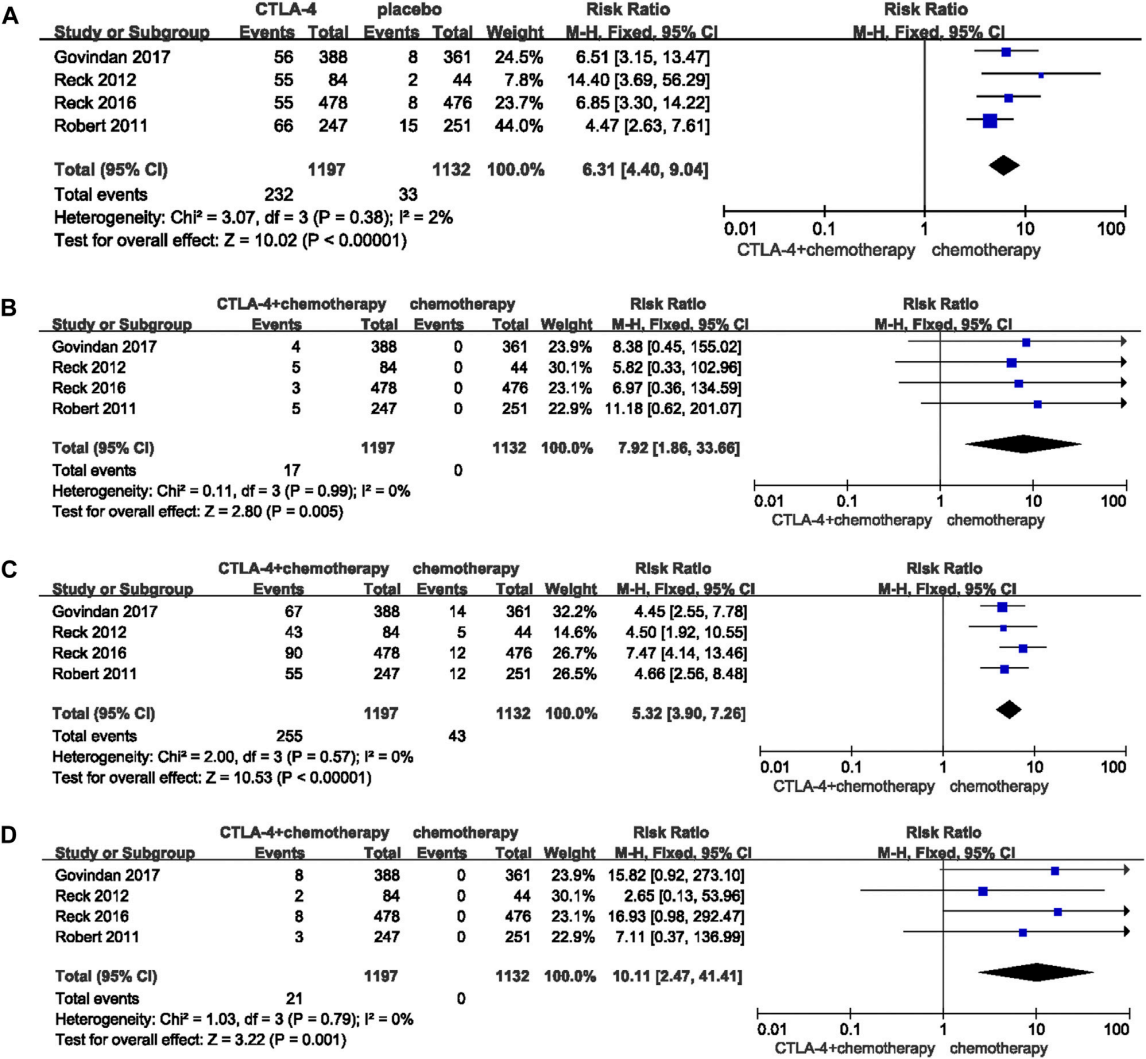
Forest plots of the relative risks and 95% CIs for pruritus and rash in comparison of CTLA-4 plus chenotherapy and chemotherapy. **(A)** any grade pruritus; **(B)** high grade pruritus; (C) any grade rash; **(D)** high grade rash.

Nine studies with 4,899 patients were suitable for this analysis. When compared with chemotherapy alone, RRs were 1.39 (95%CI 1.08-1.80, *p* = 0.01) (Figure 3A) and 1.51 (95%CI 1.25-1.83, *p* < 0.0001) (Figure 3B) for any grade pruritus and any grade rash developed after PD-1/-L1 inhibitor combined with chemotherapy. RR for high grade rash was 2.64 (95%CI 0.71-9.88, *p* = 0.15) (Figure 3C). Data was not sufficient for comparison of high grade pruritus between PD-1/-L1 plus chemotherapy and chemotherapy. Studies available included four RCTs reporting an any grade pruritus group, two of which did not report data for high grade pruritus. No patients in the remaining two studies were reported to have experienced high grade pruritus. Similarly, the combination of CTLA-4 inhibitor and chemotherapy increased the risk of pruritus and rash compared with chemotherapy [any grade pruritus RR:6.31 (95%CI 4.40-9.04, *p* < 0.00001) (Figure 4A); high grade pruritus RR:7.92 (95%CI 1.86-33.66, *p* = 0.005) (Figure 4B); any grade rash RR:5.32 (95%CI 3.90-7.26, *p* < 0.00001) (Figure 4C); and high grade rash RR:10.11 (95%CI 2.47–41.41, *p* = 0.001) (Figure 4D)].

#### Programmed Cell Death Protein 1/Programmed Cell Death-Ligand 1 Inhibitor Combined With Targeted Therapy Vs Targeted Therapy Alone

**FIGURE 5:**
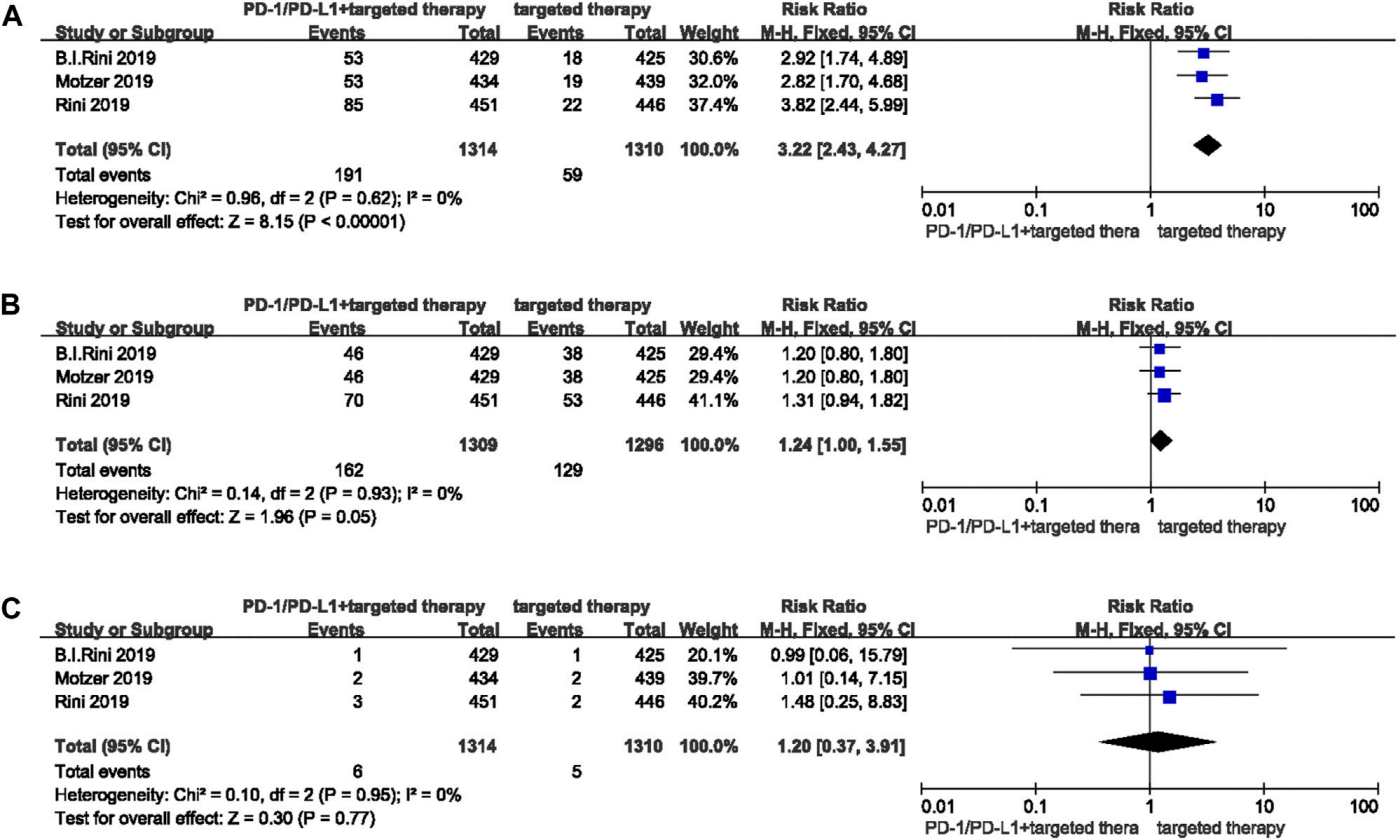
Forest plots of the relative risks and 95% CIs for pruritus and rash in comparison of PD-1/-L1 inhibitor plus targeted therapy and targeted therapy alone. **(A)** any grade pruritus; **(B)** any grade rash; **(C)** high grade rash.

Three studies with 2,624 patients were included in this section. Compared to targeted therapy, RR for any grade pruritus associated with PD-1/-L1 inhibitor combined with targeted therapy was 3.22 (95% CI 2.43-4.27, *p* < 0.00001) (Figure 5A). RRs for any grade and high grade rash were 1.24 (95% CI 1.00-1.55, *p* = 0.05) (Figure 5B) and 1.20 (95% CI 0.37-3.91, *p* = 0.77) respectively (Figure 5C).

#### Programmed Cell Death Protein 1/Programmed Cell Death-Ligand 1 and Cytotoxic T Lymphocyte Associate Protein 4 Inhibitor Combination Therapy Vs Monotherapy

**FIGURE 6:**
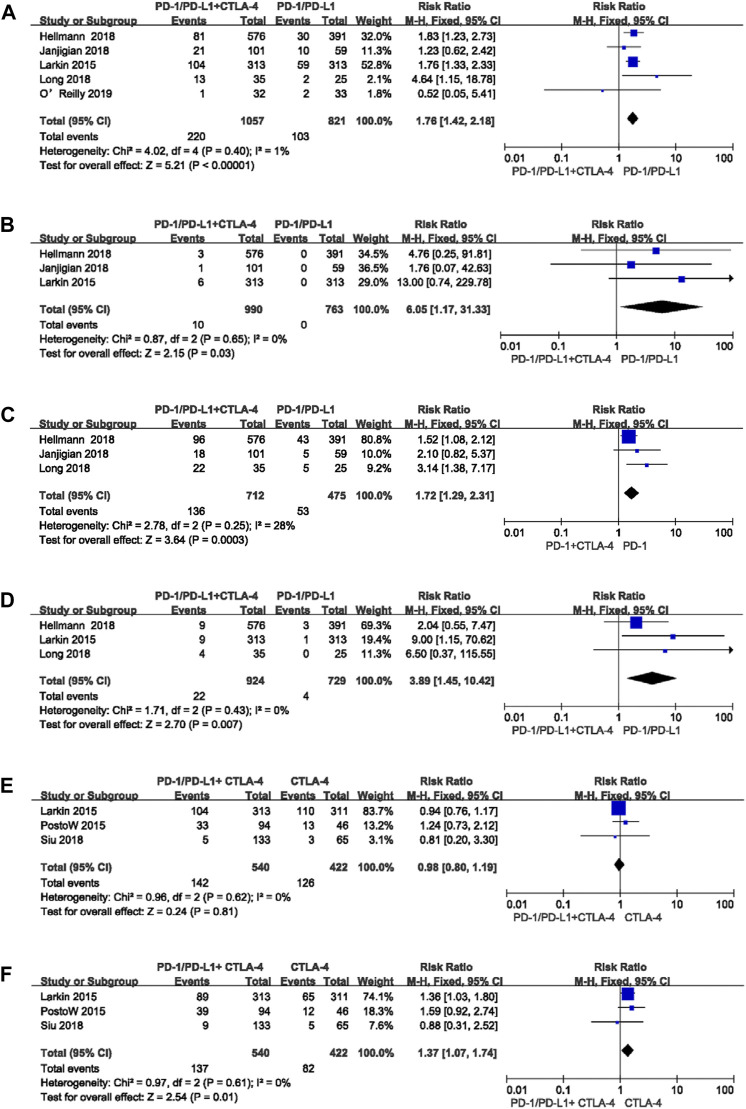
Forest plots of the relative risks and 95% CIs for pruritus and rash in comparison of combined immunotherapy and either monotherapy: **(A)** any grade pruritus for PD-1/-L1 plus CTLA-4 inhibitor compared to PD-1/-L1 inhibitor; **(B)** high grade pruritus for PD-1/-L1 plus CTLA-4 inhibitor compared to PD-1/-L1 inhibitor; **(C)** any grade rash for PD-1/-L1 plus CTLA-4 inhibitor compared to PD-1/-L1 inhibitor; **(D)** high grade rash for PD-1/-L1 plus CTLA-4 inhibitor compared to PD-1/-L1 inhibitor; **(E)** any grade pruritus for PD-1/-L1 plus CTLA-4 inhibitor compared to CTLA-4 inhibitor; **(F)** any grade rash for PD-1/-L1 plus CTLA-4 inhibitor compared to CTLA-4 inhibitor.

1,878 patients in five studies were included in the comparison between PD-1/PD-L1 plus CTLA-4 inhibitor and PD-/PD-L1 inhibitor alone. Compared to PD-1/-L1 inhibitor monotherapy, PD-1/-L1 inhibitor plus CTLA-4 inhibitor was associated with increased risk of pruritus and rash [any grade pruritus RR:1.76 (95% CI 1.42-2.18, *p* < 0.00001) (Figure 6A), high grade pruritus RR: 6.05 (95% CI 1.17-31.33, *p* = 0.03) (Figure 6B), any grade rash RR:1.72 (95% CI 1.29-2.31, *p* = 0.0003) (Figure 6C), high grade rash RR:3.89 (95% CI 1.45-10.42, *p* = 0.007) (Figure 6D)].

For comparison of PD-1/-L1 plus CTLA-4 inhibitor to CTLA-4 inhibitor monotherapy, we included four studies with 1,813 patients total. Only any grade rash was more frequent in patients administered CTLA-4 inhibitor combined with PD-1/-L1 inhibitor, in comparison to CTLA-4 inhibitor monotherapy [any grade pruritus RR:0.98 (95% CI 0.80-1.19, *p* = 0.81) (Figure 6E), any grade rash RR:1.37 (95% CI 1.07-1.74, *p* = 0.01) (Figure 6F)]. Data for high grade pruritus and high grade rash are not reported because only two studies identified included these categories, which was not sufficient for a qualified meta-analysis.

#### Programmed Cell Death Protein 1/Programmed Cell Death-Ligand 1 Inhibitor Combination Chemotherapy Vs Programmed Cell Death Protein 1/Programmed Cell Death-Ligand 1 Monotherapy or Cytotoxic T Lymphocyte Associate Protein 4 Inhibitor Combination Chemotherapy

16,039 patients from 25 studies were included in this analysis. Compared to PD-1/-L1 inhibitor monotherapy, relative risk ratios (RRRs) for any grade and high grade rash developed during PD-1/-L1 inhibitor treatment combined with chemotherapy were not significantly increased (RRR for any grade pruritus was 0.30 (95% CI 0.21-0.42, *p* < 0.00001), RRR for any grade rash was 0.84 (95% CI 0.61-1.15, *p* = 0.28), RRR for high grade rash was 1.43 (95% CI 0.46-4.40, *p* = 0.54). A comparison between PD-1/-L1 combination chemotherapy and CTLA-4 combination chemotherapy was also conducted. PD-1/-L1 plus chemotherapy was associated with decreased risk of any grade pruritus and any grade rash, compared to CTLA-4 plus chemotherapy. RRR for any grade pruritus was 0.22 (95% CI 0.14-0.49, *p* < 0.00001), RRR for any grade rash was 0.29 (95% CI 0.19–0.43, *p* < 0.00001), and RRR for high grade rash was 0.25 (95% CI 0.04-1.73, *p* = 0.08) (Table 2).

**TABLE 2:** Relative risk ratios of treatment regimen differences for the risk of pruritus and rash.

Treatment scheme	No. of trials	Any-grade pruritus		No. of trials	Any-grade rash		No. of trials	3–5 grade pruritus		No. of trials	3–5 grade rash	
		RR (95%CI)	*p*		RR (95%CI)	*p*		RR (95%CI)	RR (95%CI)		RR (95%CI)	*p*
A:PD-1/L1+chemotherapyVS chemotherapy	4	1.39 (1.08, 1.80)	0.01	5	1.53 (1.19, 1.98)	0.001	4	NA	NA	5	2.64 (0.82, 4.16)	0.15
B: PD-1/L1 VS chemotherapy	13	4.67 (3.66, 5.95)	<0.00001	12	1.82 (1.52, 2.19)	<0.00001	13	0.86 (0.28, 2.66)	0.43	12	1.85 (0.54, 2.57)	0.69
RRR (A VS B)	—	0.30 (0.21, 0.42)	<0.00001	RRR (A VS B)	0.84 (0.61, 1.15)	0.28	—	NA	NA	RRR (A VS B)	1.43 (0.46, 4.40)	0.54

Treatment scheme	No. of trials	Any-grade pruritus		No. of trials	Any-grade rash		No. of trials	3–5 grade pruritus		No. of trials	3–5 grade rash	
		RR (95%CI)	*p*		RR (95%CI)	*p*		RR (95%CI)	RR (95%CI)		RR (95%CI)	*p*
C: PD-1/L1+chemotherapy VS chemotherapy	3	1.39 (1.08, 1.80)	0.01	3	1.53 (1.19, 1.98)	0.001	3	NA	NA	3	2.64 (0.71, 9.88)	0.15
D: CTLA-4+chemotherapy VS chemotherapy	14	6.31 (4.40, 9.04)	<0.00001	14	5.32 (3.90, 7.28)	<0.00001	14	7.92 (1.86, 33.65)	0.005	14	10.11 (2.47, 41.41)	0.001
RRR (C VS D)	—	0.22 (0.14, 0.49)	<0.00001	RRR (C VS D)	0.29 (0.19, 0.43)	<0.00001	—	NA	NA	RRR (C VS D)	0.25 (0.04, 1.73)	0.08

### Subgroup Analyses

#### Programmed Cell Death Protein 1 Vs Programmed Cell Death-Ligand 1 Inhibitor

Subgroup analysis was performed to identify the relative impact of PD-1 and PD-L1 inhibitor on pruritus and rash. 20,769 patients from 42 studies were included in this analysis. Risks of any grade pruritus (RR: 1.93 (95% CI 1.40-2.67) *p* < 0.00001 Supplementary Figure 5A) and any grade rash [RR: 1.28 (95% CI 1.03-1.58) *p* < 0.00001 Supplementary Figure 5B] developed during PD-1 inhibitor therapy were decreased compared to PD-L1 inhibitor. When assessing high grade rash between PD-1 inhibitor and PD-L1 inhibitor therapies, no statistically significant difference was found [RR: 0.67 (95% CI 0.39-1.17) *p* = 0.46 Supplementary Figure 5C].

### Tumor Type Subgroup Analysis

43 studies with 24,871 patients were included in this subgroup analysis. Cancer type stratification demonstrated HNSCC has a lower risk for any grade pruritus and rash, compared to all cancer types. RRs for any grade pruritus: 1.08 (95% CI 0.26-4.38, *p* = 0.94), high grade pruritus: 0.19 (95% CI 0.01-3.94), any grade rash: 0.49 (95% CI 0.20-1.15, *p* = 0.001), high grade rash: 0.18 (95% CI 0.05-0.58, *p* = 0.004). The RRs for any grade pruritus did not reach the statistical cutoff for significance (Supplementary Figures 6A–D).

### Sensitivity Analysis and Publication Bias

Risk of bias graph and risk of bias summary are shown in Supplementary Figure 7 and Supplementary Figure 8. Sensitivity analysis showed that no single study could significantly affect the aggregated estimates (Supplementary Figure 9). However, there was mild asymmetry for RRs of pruritus and rash (Supplementary Figure 10). The Egger’s test (Supplementary Figure 11) shown some evidence of publication bias for pruritus (*p* = 0.005/*p* = 0.006) and high grade rash (*p* = 0.001), while the Begg’s test revealed no evidence of publication bias (Supplementary Figure 12).

## Discussion

With the growing number of patients receiving ICIs, there is significant need to understand associated adverse events in order to improve therapy management. In clinical practice ICIs have shown significant efficiency in multiple tumors, both as mono-and combination therapies. The unique ICI mechanism of action (Sibaud et al., 2016) is also accompanied with a series of IRAEs, which are distinguishable from traditional adverse effects of cancer treatment. Dermatological reactions, especially pruritus and rash, are some of the most common IRAEs, and can severely affect the quality of life and psychological well-being of patients (Sibaud et al., 2016). High grade rash can impact ICI treatment efficacy through dose-limiting effects or even result in treatment discontinuation (Geisler et al., 2020). To achieve better clinical efficacy, ICI combination therapy has become more commonly used. However, few studies have been conducted to assess the risk of dermatological-specific IRAEs among multiple treatment patterns. To our knowledge, the current study is the first comprehensive assessment of the relative risk of pruritus and rash among various ICI treatment regimens.

We first compared ICI monotherapy to placebo, and both PD-1/-L1 and CTLA-4 inhibitor were associated with increased risk of any grade pruritus and rash. Notably, CTLA-4 inhibitor was associated with higher risk of high grade pruritus and rash. A comparison between PD-1/-L1 inhibitor and CTLA-4 inhibitor monotherapy was also conducted. RRs for pruritus and rash developed after PD-1/-L1 inhibitor were decreased compared to CTLA-4 inhibitor, which is in line with the current mainstream consensus that CTLA-4 inhibitor is more likely to lead to pruritus and rash (Almutairi et al., 2020; Geisler et al., 2020; Hansen et al., 2017; Sibaud et al., 2016).

Whether the risk of developing pruritus and rash correlated with different dose regimens of immune checkpoint inhibitor is an important area of focus given issues regarding patient quality of life and treatment discontinuation. Previous studies have shown no significant correlation between PD-1/-L1 inhibitor dosage and incidence of pruritus and rash (Hansen et al., 2017; Robert et al., 2014). On the contrary, a retrospective study suggested that the frequency of IRAEs (pruritus and rash included) developed after Ipilimumab increased with dose. Another study reached a similar conclusion (Golian et al., 2016) that cutaneous IRAEs related to ipilimumab are dose-related. In the current study, compared with the low dose group, RRs for any grade pruritus and rash developed after PD-1/-L1 inhibitor in the high dose group were not significantly increased. The corresponding comparison between CTLA-4 inhibitor high dose and low dose group could not be carried out because of insufficient data. Overall, given the discrepancies among findings in studies assessing dose-dependencyof rash and pruritus, further efforts should be made to investigate the problem and instruct clinical application, both in terms of mechanism and clinical research.

In order to increase the percentage of patients benefiting from ICI treatment and reduce the occurrence of IRAEs, efforts are currently being made to combine current ICIs with new checkpoint inhibitors or other treatment methods to achieve synergistic effects (Kon and Benhar, 2019). In clinical practice, PD-1/-L1 and CTLA-4 inhibitor are being combined with other anti-cancer drugs including chemotherapy, targeted therapy, radiotherapy and other immunotherapies. Although traditionally regarded as immunosuppressive agents, some preclinical studies have shown that chemotherapy may have immune-stimulatory properties (Postow et al., 2015a). Some studies indicate combination chemotherapy leads to more general adverse events (Lynch et al., 2012; Wang et al., 2021), while other studies report severe side effects (Chamoto et al., 2020) . We used the relative risk ratio (RRR) to indirectly compare the risk of pruritus and rash. RRR was used to compare PD-1/PD-L1 inhibitor monotherapy with combined chemotherapy based on PD-1/PD-L1 inhibitor, and showed that the risk of pruritus, but not rash, was increased (Table 2). These results suggest that PD-1/-L1 inhibitor combined with chemotherapy may have a tolerable dermatologic adverse profile in terms of pruritus and rash, indicating that increased efficacy through combining ICIs with chemotherapy may be feasible. Targeted therapies for oncogenic signaling pathways are also attractive partners in combination with immune checkpoint blockade (Postow et al., 2015a). Unfortunately, only 2 RCTs comparing PD-1/-L1 inhibitor and targeted therapy resulted from our database search, and RRR for PD-1/PD-L1 inhibitor plus targeted therapy compared to PD-1/-L1 monotherapy could not be calculated. When more data becomes available, further analysis of this aspect may provide useful information.

Since CTLA-4 inhibitor monotherapy showed increased risk of pruritus and rash relative to PD-1/-L1 inhibitor according to our data, RRR was calculated to investigate the difference between PD-1/-L1 plus chemotherapy and CTLA-4 plus chemotherapy. When contrasted with PD-1/-L1 inhibitor combination chemotherapy, CTLA-4 inhibitor combination chemotherapy was associated with a much higher risk of pruritus and rash (Table 2). The mechanism leading to this is not yet fully understood. The major physiological role of CTLA-4 seems to be through distinct effects on the two main subsets of cluster of differentiation four positive (CD4^+^) T cells: down modulation of helper T cell activity and enhancement of regulatory T (Treg) cell immunosuppressive activity (Bylicki et al., 2020; Cancela et al., 2020; Peggs et al., 2009). Blockade of the PD-1 pathway may enhance antitumor immune responses by diminishing the number and/or suppressive activity of intratumoral Treg cells (Arigami et al., 2020). It is thought that PD-1 predominantly regulates effector T cell activity within tissue and tumors, whereas CTLA-4 predominantly regulates T cell activation (Arigami et al., 2020). Although dermatologic adverse events observed with ICIs used in combination are more frequent, more severe, and longer lasting (Sibaud et al., 2016), combination immunotherapy has more extensive clinical applications due to improved efficacy. Therefore, our data suggest that PD-1/-L1 inhibitor may be preferable in patients who have suffered from previous dematologic problems. Moreover, in the case of severe dermatologic IRAEs with CTLA-4 therapy, re-challenge with an agent of a different class may be a good treatment strategy.

Subgroup analysis was performed to investigate if there was any difference in the incidence of pruritus and rash between PD-1 and PD-L1 inhibitor. Based on the known interactions of PD-1 ligands, PD-1 antibodies may have different biological activities than PD-L1 antibodies. PD-1 antibodies prevent PD-1 from interacting with PD-L1 and Programmed cell death-ligand 2(PD-L2), but do not prevent the interaction between PD-L1 and Cluster of differentiation 80(CD80). In contrast, most PD-L1 antibodies prevent the interaction between PD-L1 and CD80 and between PD-L1 and PD-1, but not the interaction between PD-1 and PD-L2. Therefore, it is possible that depending on which interaction predominates in a particular cancer, PD-1 and PD-L1 antibodies may not have redundant activity (Arigami et al., 2020). Results from subgroup analysis showed that any grade pruritus and rash developed from PD-1 inhibitor were decreased compared to PD-L1 inhibitor, while the comparison in high grade (3–5) rash did not reach a statistically significant level. Therefore, PD-1 inhibitor may be recommended in terms of decreased dermatologic adverse events (pruritus and rash) for clinical applications. In cancer type subgroup analysis, we found that patients with HNSCC may have better tolerability overall as evidenced by a lower risk for any grade pruritus. Since only 1 RCT of HNSCC was included in high grade subgroup, more efforts are needed to validate this observation.

Our study has some notable strengths. To the best of our knowledge, this is the first and most comprehensive analysis that investigated the risk of pruritus and rash among different ICI treatment regiments in multiple solid tumors. In addition, the 50 clinical trials included in our meta-analysis were all highly qualified randomized control trails, which supports the credibility of our study. Morever, we investigated the risk of not only all grade but also high grade pruritus and rash, for the management of these two side effects of differing severity. Finally, since head-to-head comparison of PD-1/PD-L1 inhibitor combination therapies and PD-1/PD-L1 inhibitor alone were not available, we used the relative risk ratio (RRR) to indirectly compare the risk of pruritus and rash. The results of our RRR analysis indicate that the added skin toxicity of chemotherapy is manageable in combination immunotherapy, which may have clinical implications.

This meta-analysis also has some limitations. Mild heterogeneity was found among the included studies. The heterogeneity may result from differences in cancer type, line of therapy, follow-up time, or other unspecified factors. Study design, blinding, dosage and frequency of drug administration in both intervention and control arm could also have resulted in heterogeneity. Thus, we utilized the random-effect model and subgroup analyses for high heterogeneity to explore possible variation in the outcomes of the included studies. What`s more, since patients included in our meta-analysis were from RCTs with strict inclusion criteria, risk of pruritus and rash could be underestimated because of their better health condition, compared with patients in real world application.

## Conclusion

In summary, we identified that PD-1/-L1 inhibitor is associated with decreased risk of pruritus and rash in comparison to CTLA-4 inhibitor in both monotherapy and combined immunotherapy regimens. Additionally, pruritus and rash developed from PD-1/-L1 inhibitor are not dose-dependent. Moreover, compared to PD-1/-L1 inhibitor alone, the combination of chemotherapy with PD-1/-L1 inhibitor may not significantly increase the risk of pruritus and rash. As the most prevalent and obvious IRAEs, dermatologic adverse events such as rash and pruritus should be further studied to help manage such events and enhance patient benefits from ICI therapy.

## Data Availability

The original contributions presented in the study are included in the article/Supplementary Material, further inquiries can be directed to the corresponding authors.
