# Supplementary material for: Differential Dermatologic Adverse Events Associated With Checkpoint Inhibitor Monotherapy and Combination Therapy: A Meta-Analysis of Randomized Control Trials
Source: Front Pharmacol. 2021 Jul 22;12:640099. doi: 10.3389/fphar.2021.640099 (PMC8383780; doi:10.3389/fphar.2021.640099)
Supplement: Supplementary file 1 [file datasheet1.docx]

Supplementary material for

**Differential** **dermatologic adverse events associated with checkpoint inhibitor monotherapy and combination therapy: a meta-analysis of randomized control trials**

Yang Ge^1†*^, Hui-Yun Zhang ^1†^, Nathaniel Weygant^2,3^ , Jiannan Yao^1*^

^1^Beijing Chao-Yang Hospital, Capital Medical University, Dept. of Oncology, Beijing, CN

^2^Fujian Univ. of Traditional Chinese Medicine, Academy of Integrative Medicine, Fuzhou, Fujian, CN

^3^Fujian Key Laboratory of Integrative Medicine in Geriatrics, Fuzhou, Fujian, CN

***Correspondence：**

Yang Ge

Interna-1@163.com(Y.G)

Jiannan Yao

silversand1986@sina.com

**Supplementary Figure 1.** Forest plots showing the relative risks and 95% CIs for pruritus and rash in comparison between PD-1/-L1 inhibitor and placebo. (A) any grade pruritus.(B) any grade rash. (C) high grade rash.

**
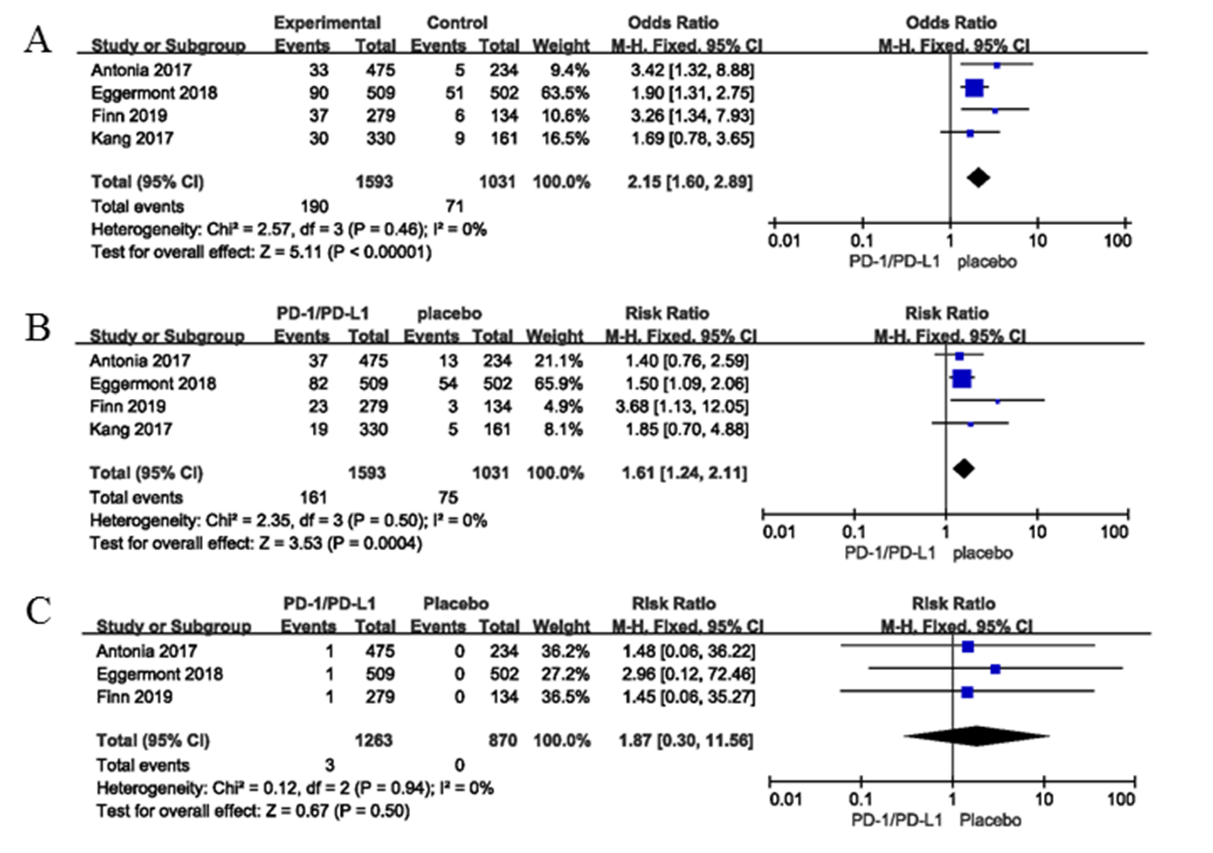
**

**Supplementary Figure 2.** Forest plots showing the relative risks and 95% CIs for pruritus and rash in comparison between CTLA-4 inhibitor and placebo. (A) any grade pruritus. (B) high grade pruritus.(C) any grade rash. (D) high grade rash.

**
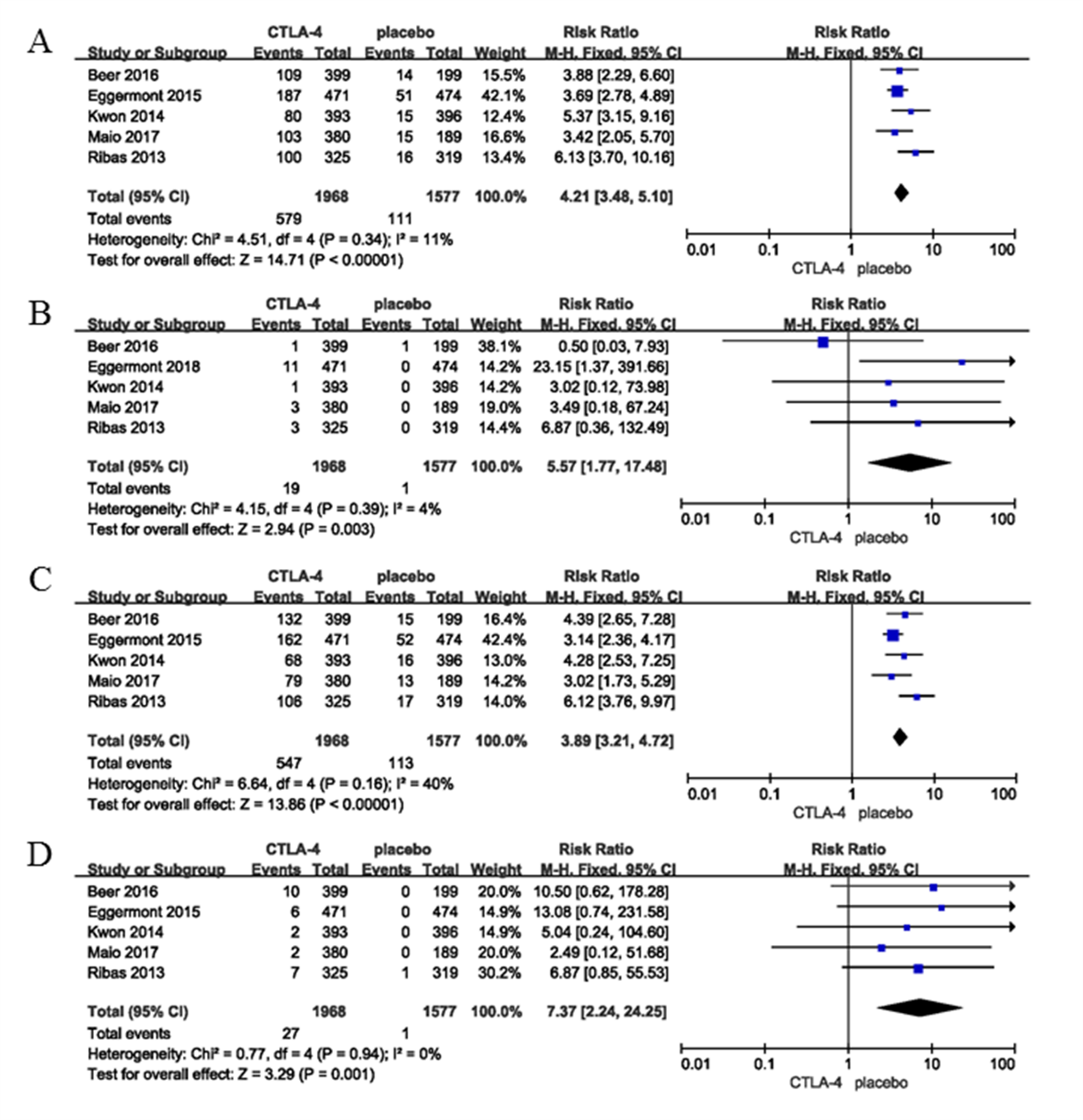
**

**Supplementary Figure 3.** Forest plots showing the relative risks and 95% CIs for pruritus and rash in comparison between PD-1/-L1 inhibitor and CTLA-4. (A) any grade pruritus. (B) high grade pruritus.(C) any grade rash. (D) high grade rash.

**
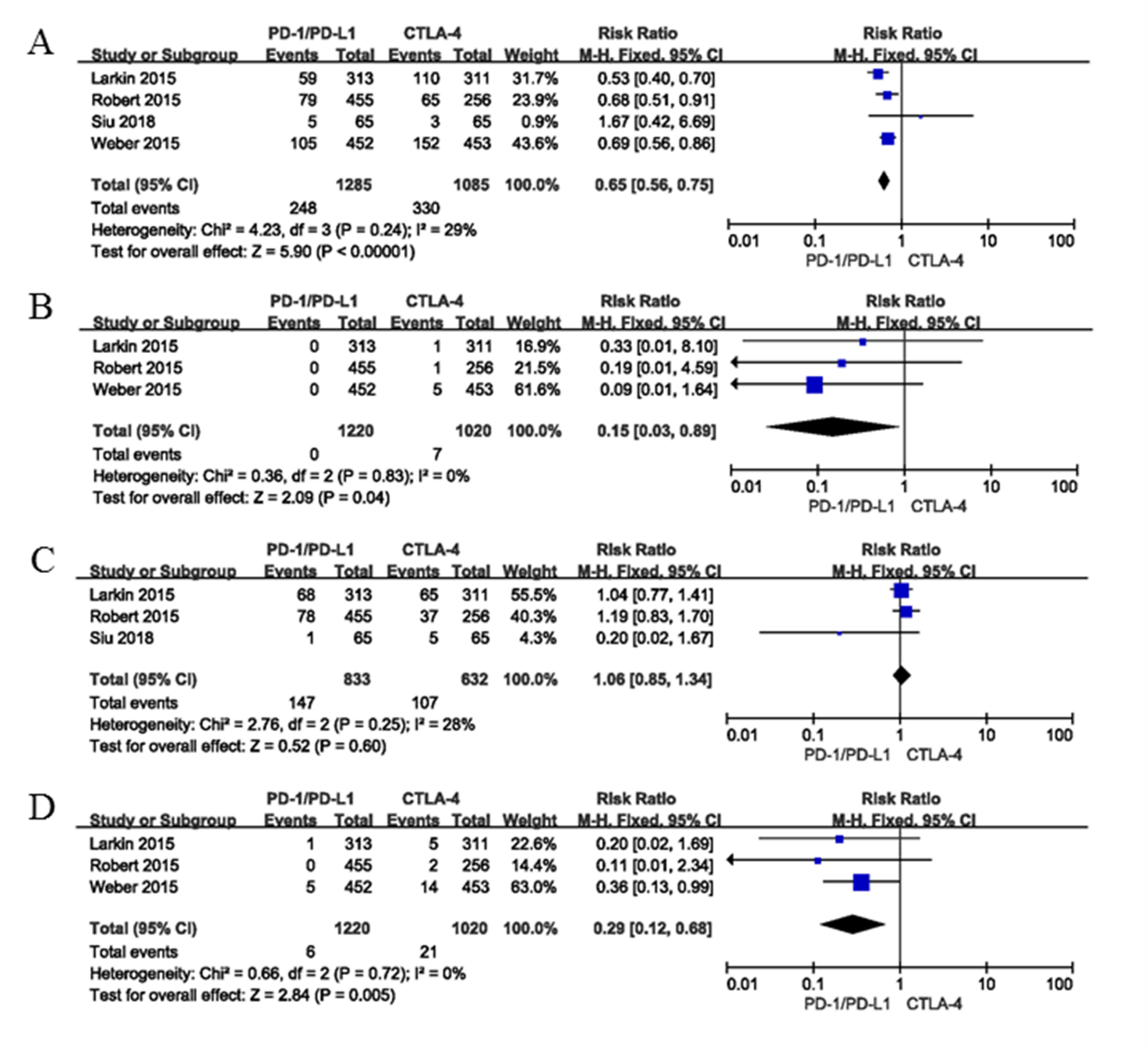
**

**Supplementary Figure 4.** Forest plots of (A) any grade pruritus and (B) any grade rash in comparison of high dose VS low dose.

**
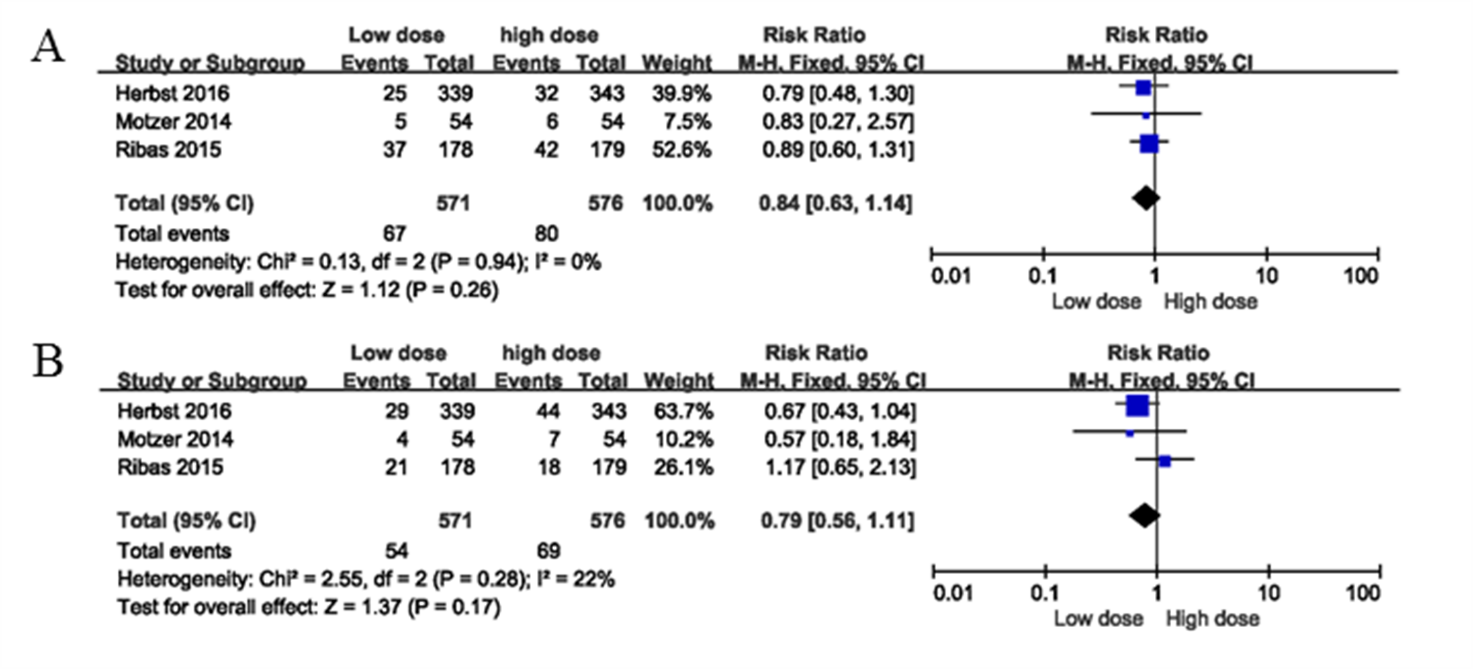
**

**Supplementary Figure 5.**Subgroup analysis of risks for any grade pruritus (A), any grade rash (B) and high grade rash (C) between experimental arm and control arm, according to class of ICIs.

**

**

**

**

**

**

**Supplementary Figure 6:** Subgroup analysis of risks for any grade pruritus (A), high grade pruritus (B), any grade rash (C) and high grade rash (D) between experimental arm and control arm, according to cancer type.

**
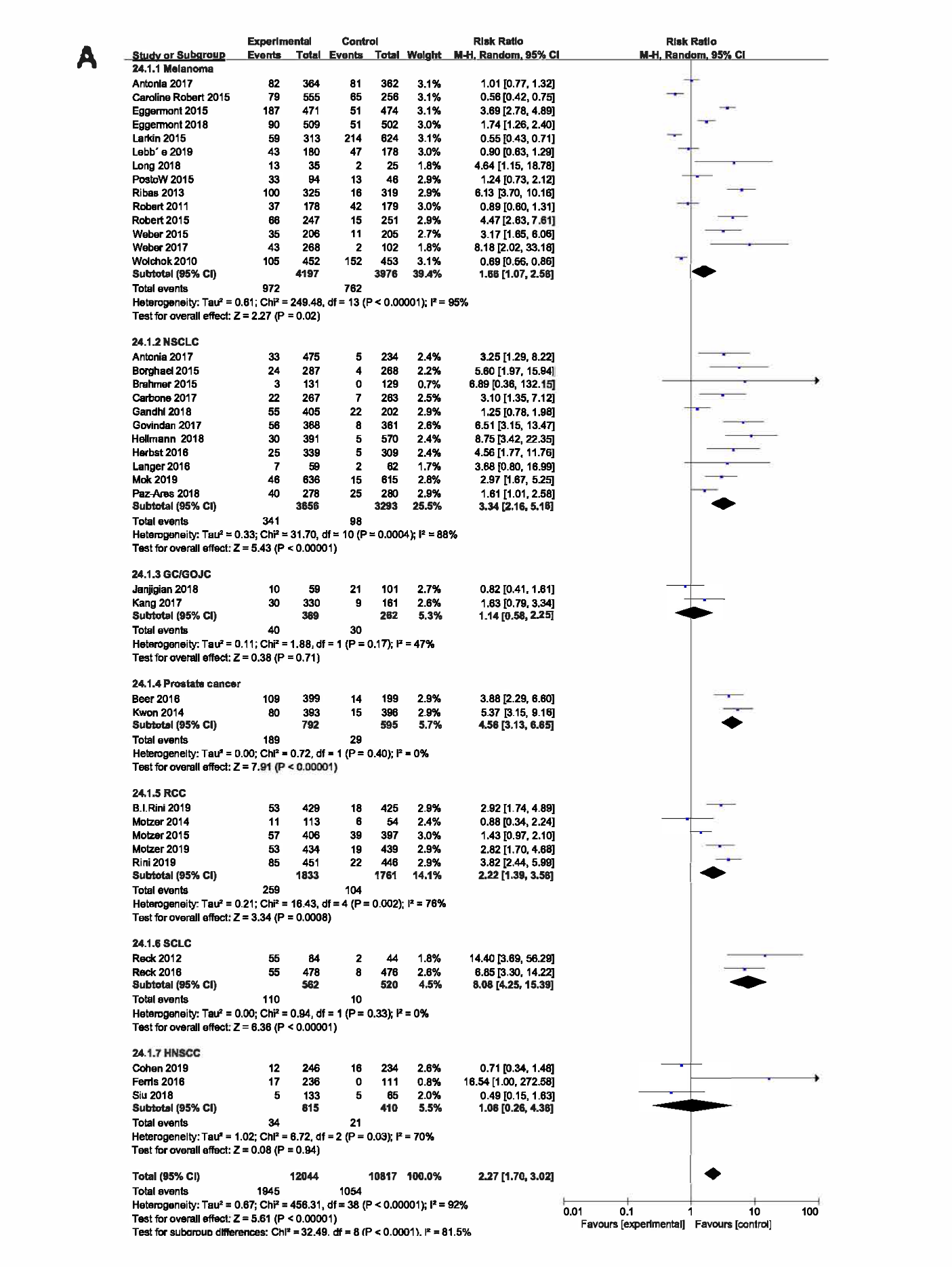
**

**
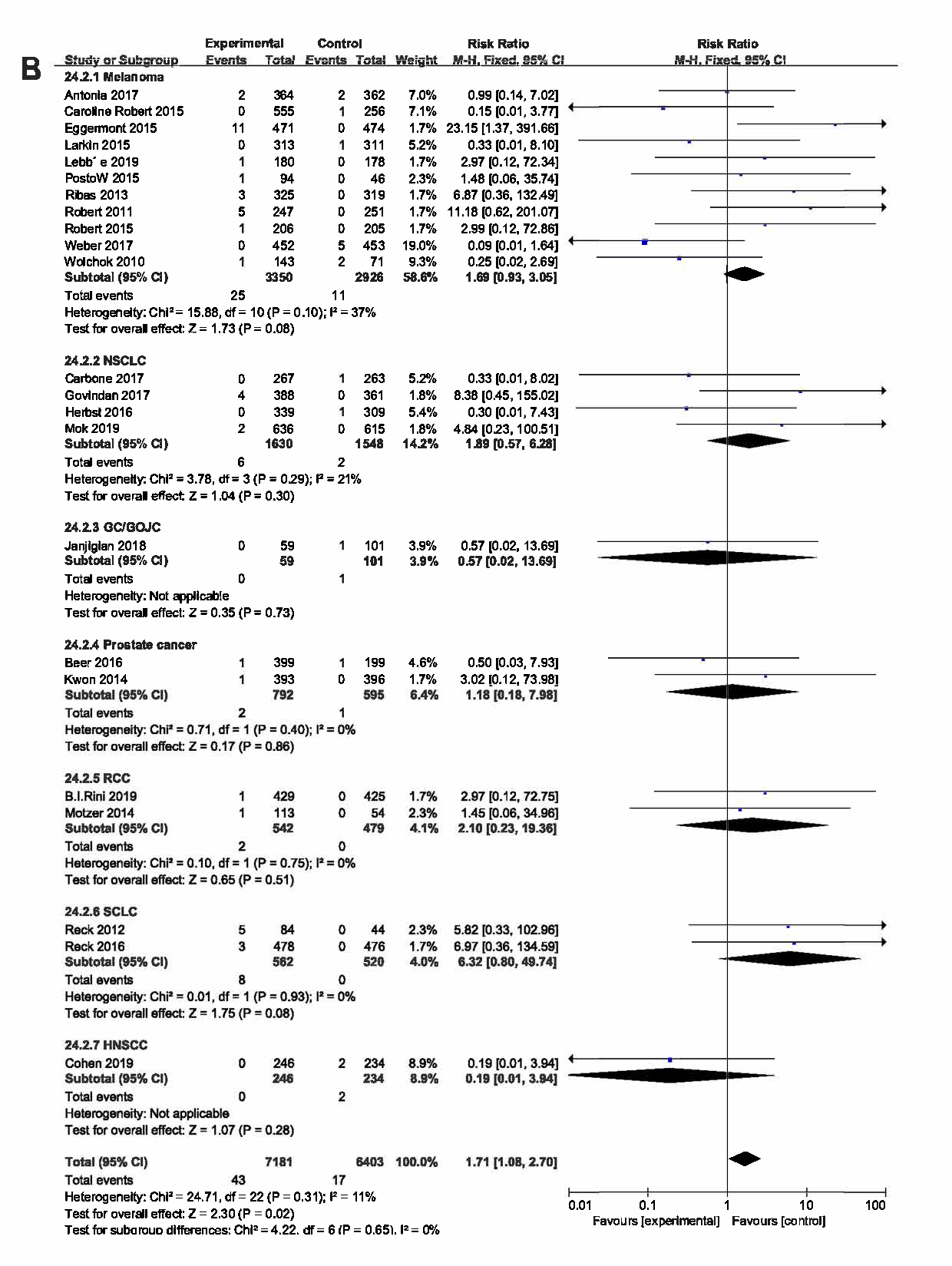
**


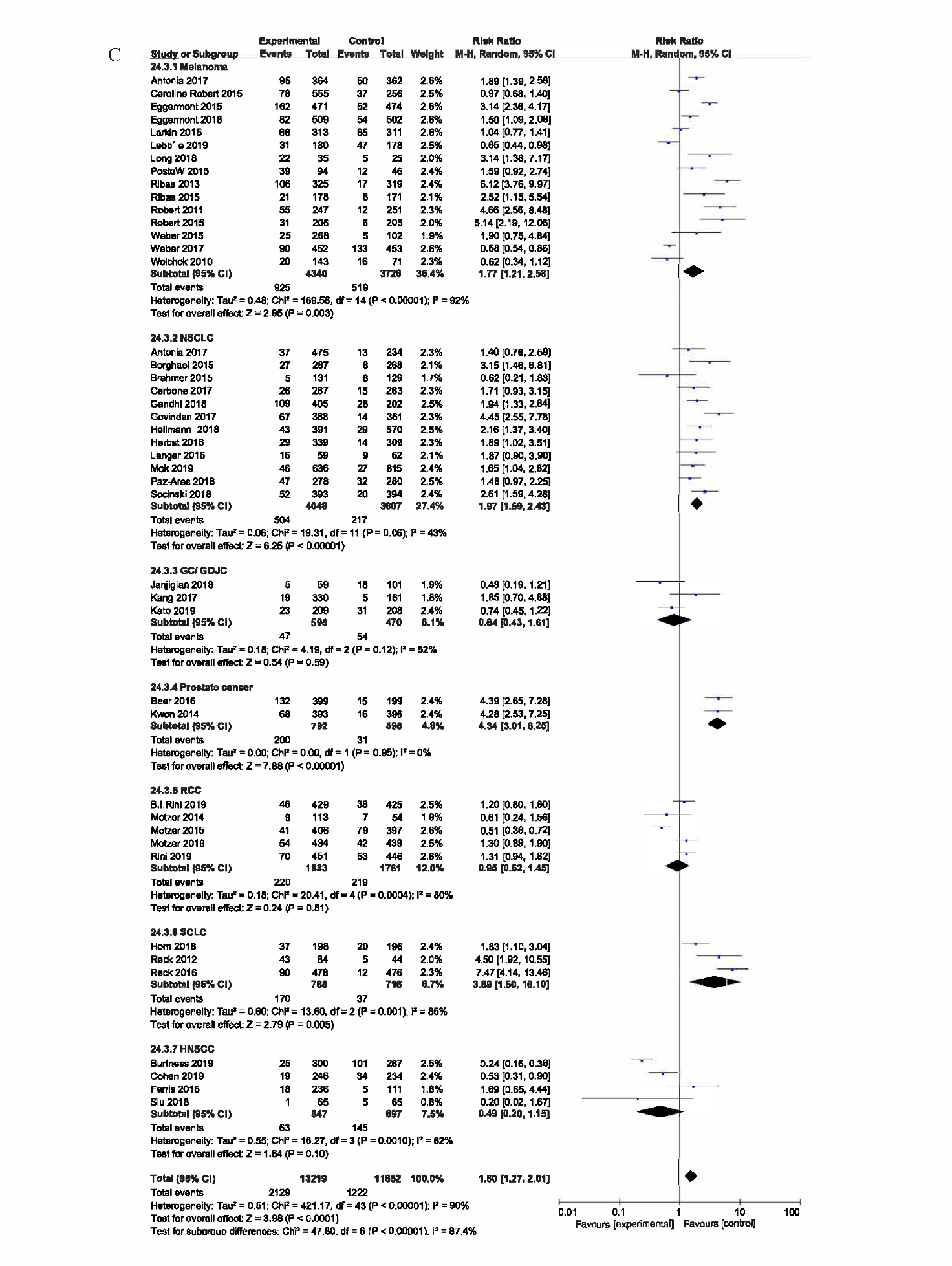


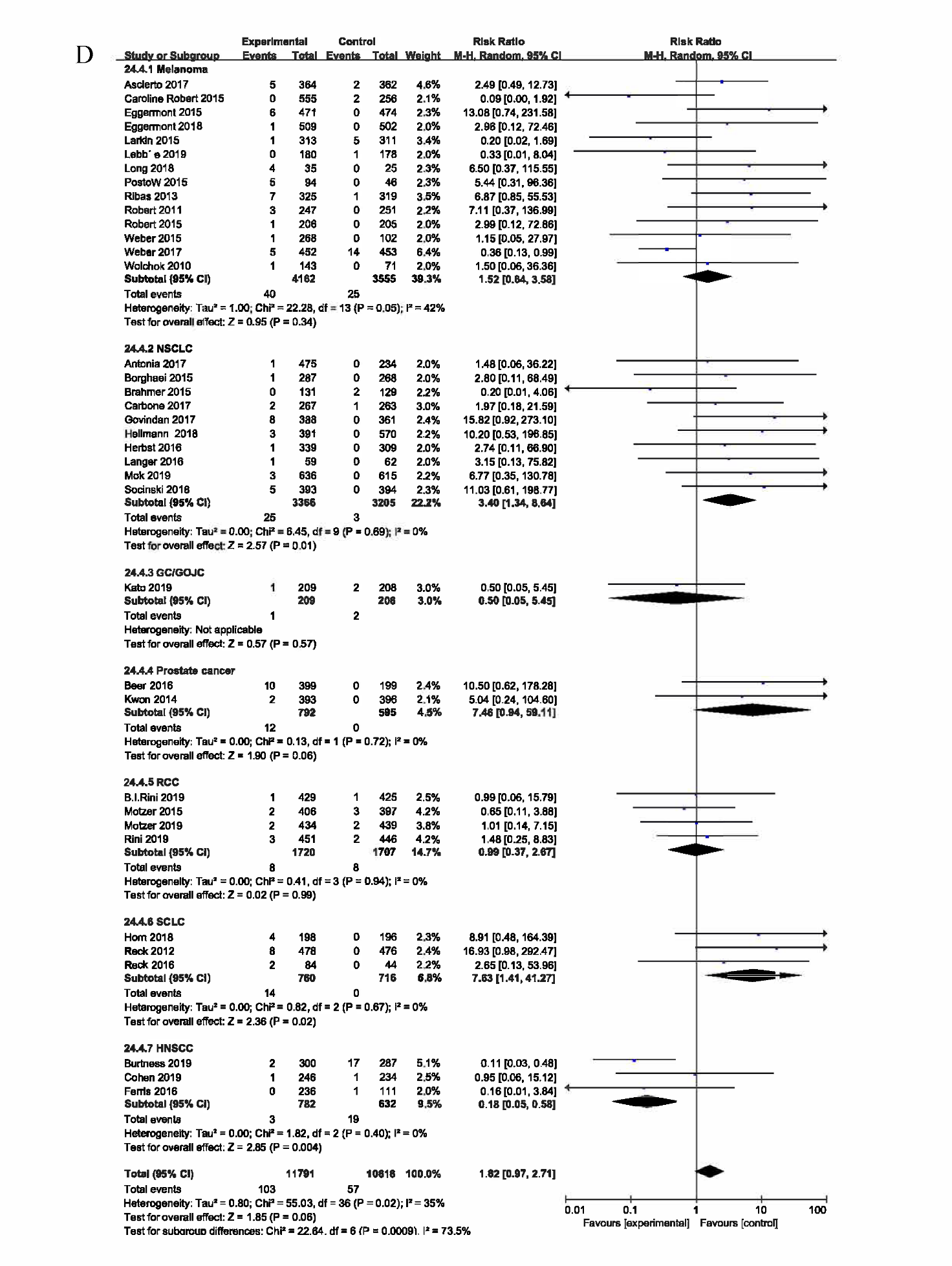


**Supplementary Figure 7:** Risk of bias graph: evaluated according to the Cochrane Collaboration recommendation.


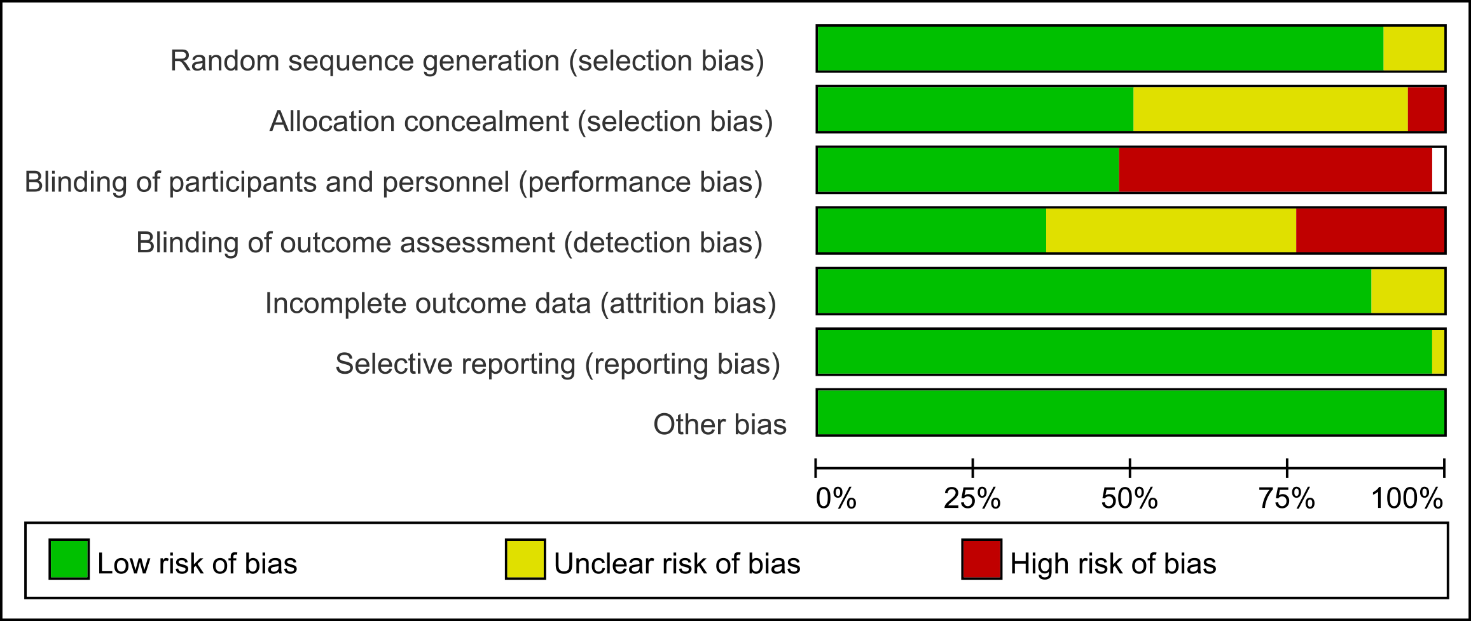


**Supplementary Figure 8.** Risk of bias summary: Judgments from review authors’ about each risk of bias item for each selected study.


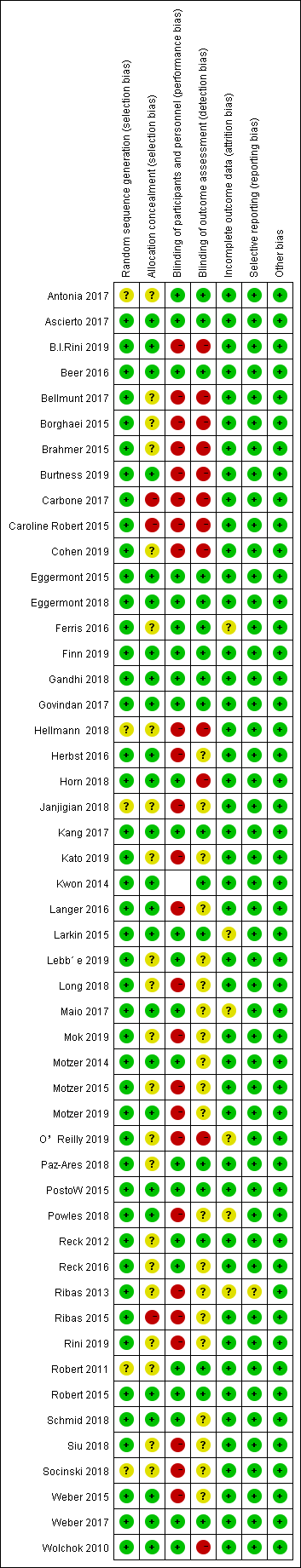


**Supplementary Figure 9:** Sensitivity analysis of any grade pruritus (A), high grade pruritus (B), any grade rash (C) and high grade rash (D).


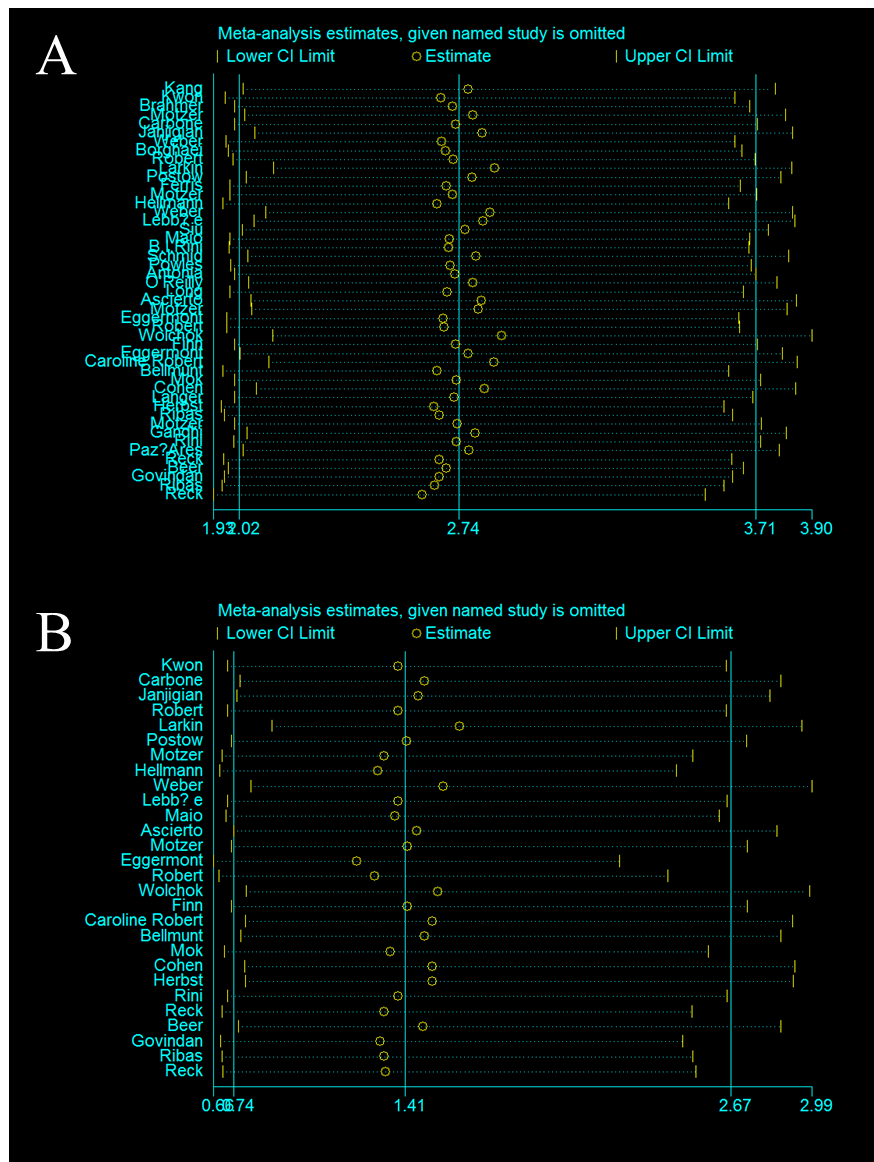


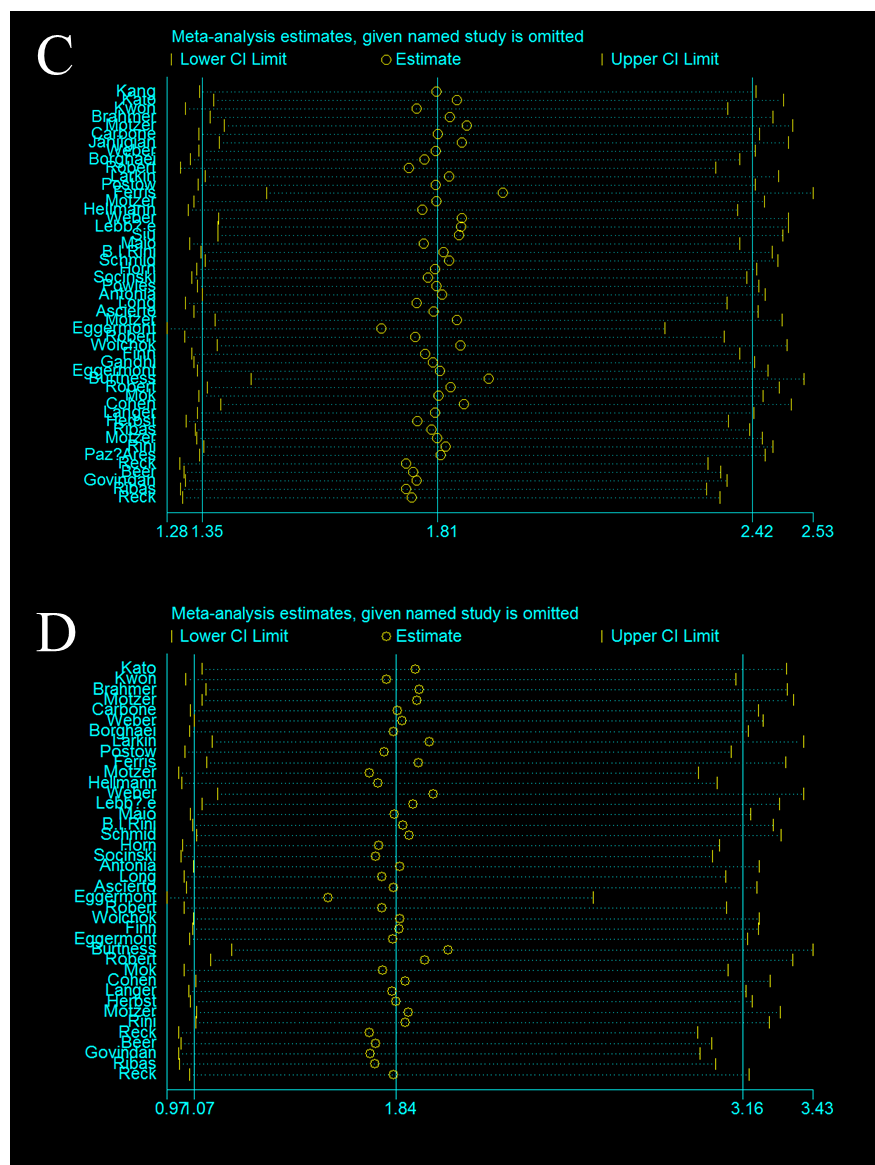


**Supplementary Figure 10:** Funnel plots of any grade pruritus (A), high grade pruritus (B), any grade rash (C) and high grade rash (D).


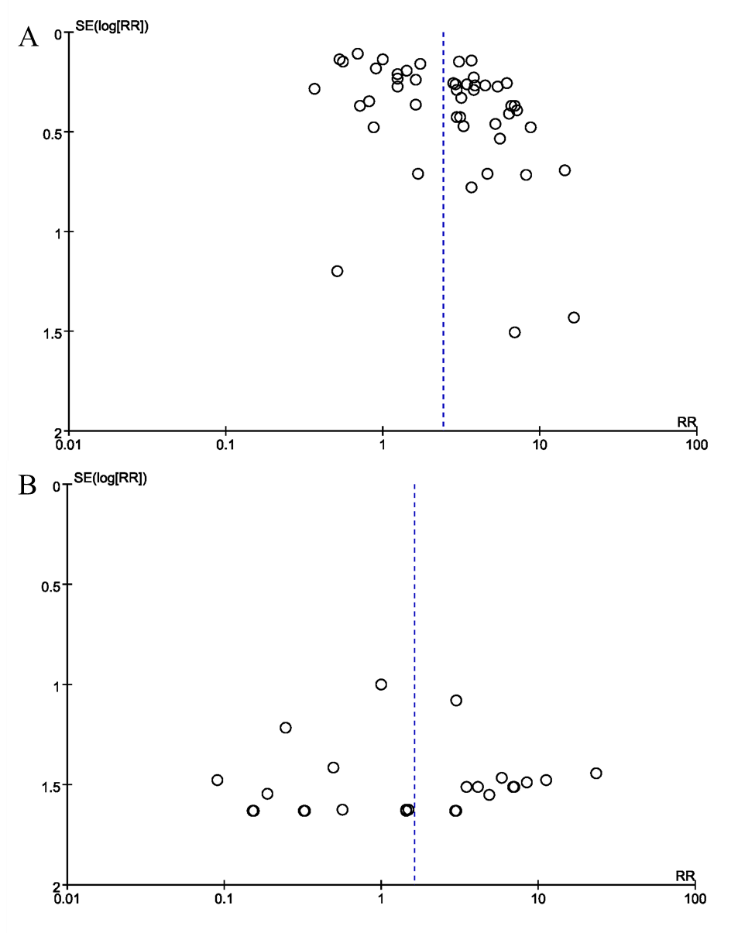

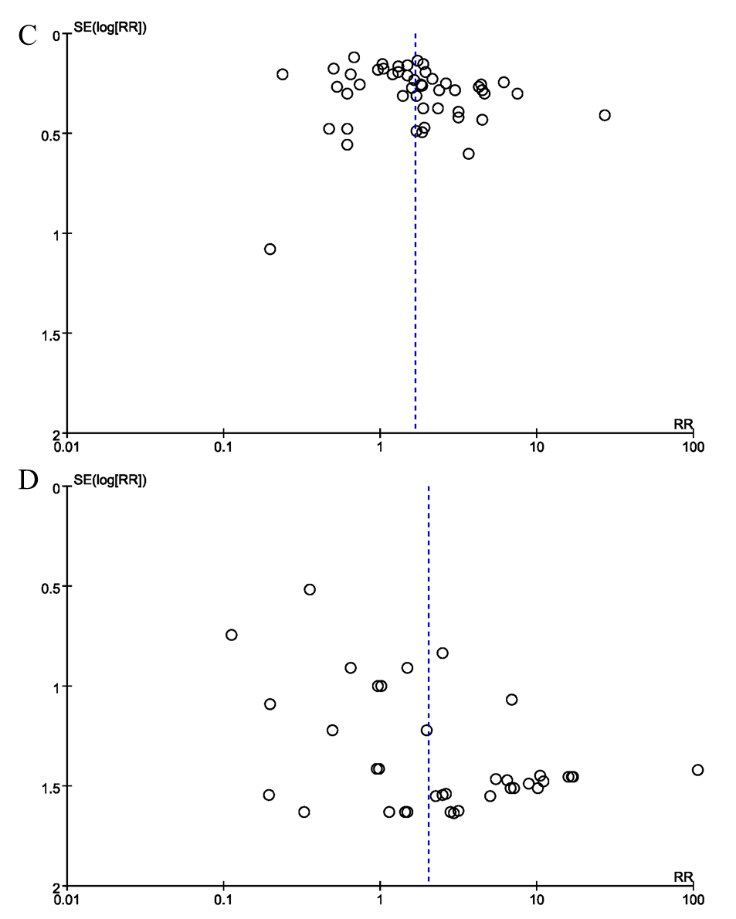


**Supplementary Figure 11：**Egger’s test for any grade pruritus (A), high grade pruritus (B), any grade rash (C) and high grade rash (D).

**
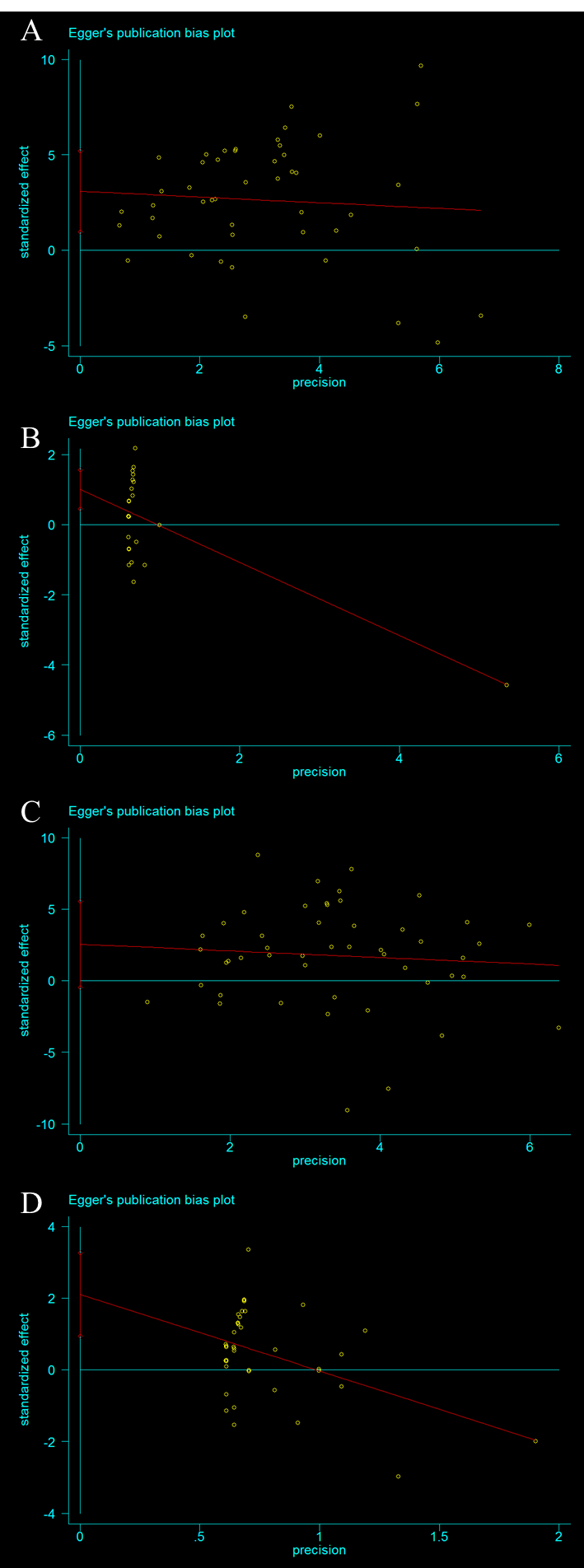
**

**Supplementary Figure 12：**Begg’s test for any grade pruritus (A), high grade pruritus (B), any grade rash (C) and high grade rash (D).

**
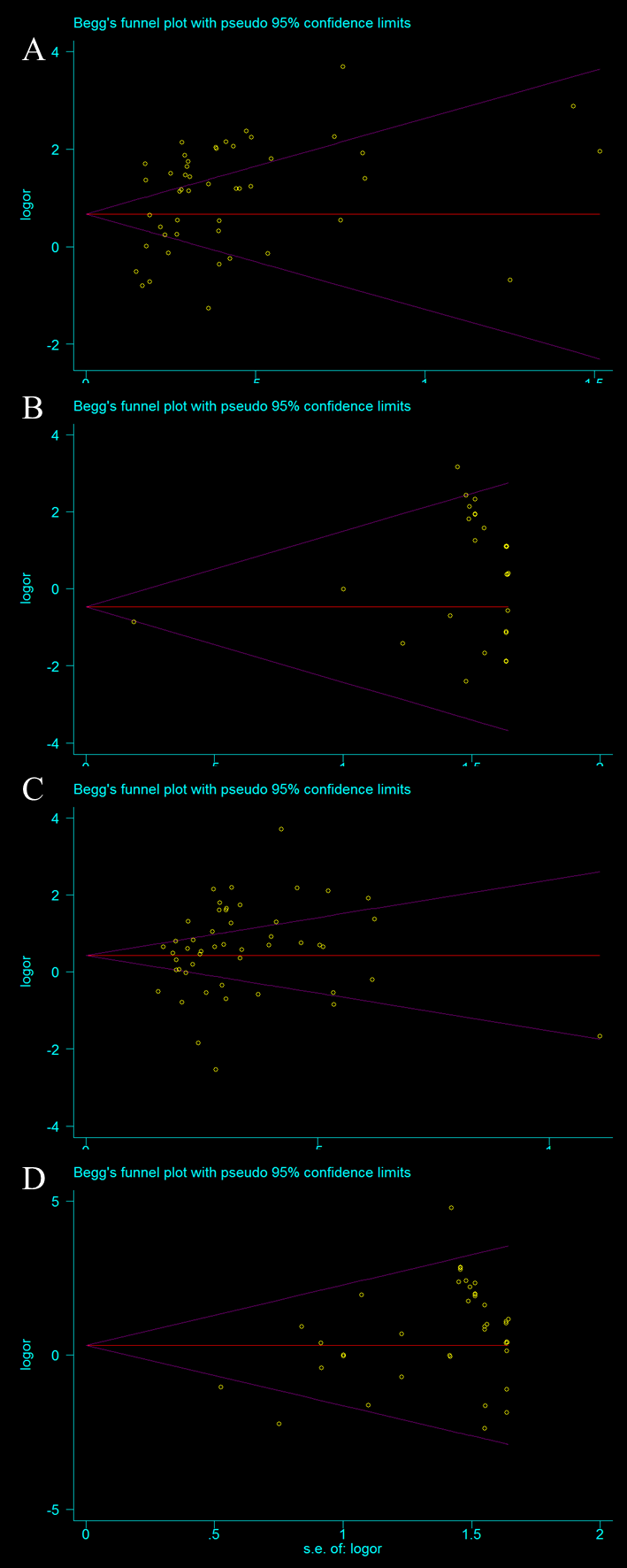
**
